# Peripheral complement C4 protein in schizophrenia: Association with gene copy number and immune cell subtypes

**DOI:** 10.1073/pnas.2536376123

**Published:** 2026-05-11

**Authors:** Agnieszka Kalinowski, Claudia Macaubas, Hanmin Guo, Lauren A. Anker, Diane E. Wakeham, Marcus Ho, Reenal Pattni, Batuhan Bayram, Surbhi Sharma, Joanna Liliental, Jong H. Yoon, Elizabeth D. Mellins, Lawrence Steinman, Alexander E. Urban

**Affiliations:** ^a^Department of Psychiatry and Behavioral Sciences, Stanford University, School of Medicine, Stanford, CA 94305; ^b^Department of Pediatrics, Stanford University, School of Medicine, Stanford, CA 94304; ^c^Department of Genetics, Stanford University, School of Medicine, Stanford, CA 94304; ^d^Translational Applications Service Center, Stanford University, School of Medicine, Department of Medicine, Division of Research and Education, Stanford, CA 94305; ^e^Translational Research and Applied Medicine Center, Stanford University, School of Medicine, Department of Medicine, Division of Research and Education, Stanford, CA 94305; ^f^Repetitive Transcranial Magnetic Stimulation Neuromodulation Clinic, Palo Alto Veterans Healthcare System, Palo Alto, CA 94304; ^g^Department of Neurology and Neurological Sciences, Stanford University, School of Medicine, Stanford Multiple Sclerosis and Neuroimmunology Program, Stanford, CA 94305

**Keywords:** schizophrenia, neutrophil, monocyte, complement C4, innate immunity

## Abstract

The lack of highly effective disease-modifying treatments for schizophrenia necessitates exploration of novel aspects of its pathophysiology, including attention to innate immune mechanisms outside the brain. C4 protein activation, associated with the complement cascade of innate immunity, associates with symptoms and predicts outcomes in schizophrenia. However, C4 protein activation does not coincide with expected changes to other proteins in the complement cascade, suggesting another source of C4 protein activation. Studying a combination of fresh whole blood from 10 anonymous donors and a large set of publicly available microarray data, we show that C4 protein is found and expressed primarily in neutrophils and monocytes. Then, we compared the correlation between C4 protein in neutrophils, classical monocytes, plasma, and the number of C4A gene copies. We determined the number of C4A genes using digital droplet PCR, C4 protein in neutrophils (15 patients/21 controls) and plasma (30 patients/38 controls) using Western blotting, and classical monocytes (30 patients/38 controls) using flow cytometry. We found a large positive correlation between the number of C4A gene copies and the amount of C4 protein only in neutrophils and only in the schizophrenia group (Spearman’s rho = 0.63, 95% BCa CI: 0.12 to 0.89, *P* = 0.012). Our results indicate a convergence of innate immunity mechanisms associated with schizophrenia. The involvement of innate immunity deserves further attention to determine whether it could be a target for therapy in schizophrenia.

## Significance

The number of C4A gene copies is associated with the risk of schizophrenia in genome-wide association studies of individuals with European ancestry. Higher C4A gene expression is associated with higher levels of synaptic pruning in the brain. We found that neutrophils from people with schizophrenia show C4 protein amounts that are positively correlated with the number of C4A gene copies. Neutrophils may gain access to the central nervous system, during some critical periods in the development of schizophrenia. The role of neutrophils both outside the brain in the peripheral circulation and within the brain invites further exploration, potentially leading to new therapeutics.

Given the lack of disease-modifying agents to treat schizophrenia (SZ), understanding its pathophysiology is imperative. Convergent evidence from epidemiological, animal, and clinical studies points to innate immune system activation, often referred to as the Innate Immune Hypothesis, as an important element of the disease mechanism in SZ (1–3). Large epidemiological studies have shown that infections in early childhood that stimulate the innate immune response are significant risk factors for SZ (4, 5). The number of neutrophils and monocytes, which are key cells of the innate immune system, are increased in people experiencing their first episode of psychosis (FEP) and chronic SZ (6). Innate immune activators, such as cytokines and proteins involved in the complement cascade, are elevated in both the serum and cerebrospinal fluid (CSF) of individuals with chronic SZ, FEP, and in the serum of individuals at clinical high risk (CHR) for psychosis (3, 7–12). These circulatory immune factors have been associated with neuroimaging findings, symptoms, and cognitive measures in SZ (13, 14), and predict clinical outcomes in CHR and SZ (15, 16).

Understanding the key pathological mechanisms of innate immunity in SZ may facilitate identification of novel therapeutic targets. The relevance of peripheral immune mechanisms to brain functioning has been demonstrated (17, 18). Modification by infusing plasma from younger subjects in the peripheral circulation improves cognition in Alzheimer’s disease in animal and in human studies (19, 20). However, clinical trials investigating drugs targeting the innate immune response in schizophrenia have yielded inconsistent results (21–23). These mixed outcomes underscore the need to better understand how the innate immune system might be altered in SZ.

A consistent finding of complement activation in individuals at risk for and with SZ, specifically involving C4 protein, offers a promising line of investigation (10, 11, 15, 24). C4 protein comes in two forms: C4A and C4B forms of the C4 protein, expressed by the C4A and C4B genes, respectively. The number of C4A gene copies is associated with the risk of SZ in genome-wide association studies (GWAS) of European ancestry (25). Higher C4A gene expression has been associated with higher levels of synaptic pruning in animal and in vitro induced-pluripotent stem cell studies (26, 27), suggesting a link between C4A gene copies and excessive synaptic pruning. This Excessive Pruning hypothesis is further supported by the finding of higher C4A gene expression in postmortem brain tissue and elevated C4A protein in CSF in individuals in FEP who develop SZ (25, 28, 29). While the evidence points to C4A gene mechanisms in the brain, emerging data suggest that they may also be important in the periphery (10, 11, 24, 30). In plasma, C4 protein is part of the classical or lectin pathway of the complement cascade, an arm of innate immunity (24). Upon activation, C4 protein is enzymatically cleaved to produce an activation product, C4-anaphylotoxin (C4-ana) and a cleaved C4 protein that binds other proteins in the complement cascade. Measuring the concentrations of activation products and cleaved proteins along parts of the complement cascade allows us to map which portion of the complement cascade is active. SZ clinical studies consistently find increased concentrations of C4-ana and decreased concentrations of its inhibitor, C4-BP, (which suggests high utilization) in plasma samples from individuals at risk for and with SZ (11, 12, 15, 24, 30). However, clinical studies have not found consistent evidence of a corresponding decrease in C4 protein concentration or changes in the concentrations of accompanying proteins along a specific pathway of the complement cascade in SZ samples (30–32). This inconsistency suggests that C4 protein is responsible for the increased concentration of C4-ana in SZ samples, which may originate from a nonplasma source in the peripheral circulation. Complement proteins in the plasma are produced in the liver (33, 34). However, recent findings have revealed that some complement proteins are both expressed and activated inside distinct immune cell types (35). This led us to ask whether 1) C4 protein could be produced and activated in/on distinct immune cell types (neutrophils and monocytes), and 2) whether the amount of C4 protein in neutrophils and/or monocytes is decreased because of C4 protein activation in SZ samples compared to controls.

To test our hypothesis that the C4 protein in neutrophils and monocytes is activated in SZ, we conducted a two-part study. In part one, to establish whether C4 protein is expressed in neutrophils and/or monocytes, we used publicly available gene expression data and validated which immune cells contain C4 protein using whole blood samples from a small cohort of anonymous blood donors. These studies allowed us to confirm that C4 protein is expressed and present in neutrophils and monocytes. In the second part of our study, we compared the amount of C4 protein (as a function of the number of C4A gene copies) in neutrophil and monocyte samples from individuals with early SZ and controls. We expected C4 protein to be expressed and consumed via C4 protein activation in the setting of innate immune system activation in SZ. Therefore, we reasoned that examining the correlation between cell-associated C4 protein and the number of C4A gene copies may more accurately reflect the dynamic process of active C4 protein expression and activation/consumption. Similarly, in our previous study, we found a preliminary association between C4 protein activation (plasma C4-ana concentration) and the number of C4A gene copies (24). Finally, we conducted exploratory analyses comparing the amount of C4 protein in different immune cell populations in samples from SZ and controls, and whether they correlate with SZ-symptom measures or stress.

## Methods

### Unbiased Examination of C4 Protein in Immune Cell Types Using Fresh Whole Blood.

To determine which immune cell types contain C4 protein in an unbiased manner, we collected fresh whole blood samples from volunteers who donated blood to the Stanford Blood Center on the day we performed the experiment. Whole blood was collected by venipuncture in 6 mL K2 EDTA blood collection tubes and kept at room temperature. Whole blood samples were stained with antibodies against the major immune cell subtypes: CD3, CD14, CD16, CD19, CD45, CD56, CD66b, and HLA-DR (*SI Appendix*, Table S2 contains details of flow cytometry antibodies used). Red blood cells were then lysed, and white blood cells were fixed using the eBiosciences 1-step Fix/Lyse solution (Invitrogen, Waltham, MA) according to the manufacturer’s instructions. White blood cells were collected by centrifugation (500× g for 5 min at room temperature). To access both surface and intracellular C4 protein, we permeabilized white blood cells by resuspending the centrifuged cell pellet in Permeabilization Buffer according to manufacturer instructions (eBioscience Permeabilization Buffer, Invitrogen). Then, cells were resuspended in permeabilization buffer and an antibody against the C4 protein beta chain (identical targets in both the A and B forms of C4 protein) with a conjugated fluorescent tag (bs-151186R-BF647, Bioss, Woburn, MA) for 30 min at room temperature with rotation in the dark. The cells were washed with FACS buffer (1% bovine serum albumin and 0.1% sodium azide in phosphate-buffered saline, pH 7.2) and centrifuged (500× g for 5 min at room temperature). Flow cytometry was performed using a Symphony Instrument at the Stanford Shared FACS Facility with the following detectors: B780, B515, V710, V610, U379, R780, R670, Y586, and Y670. All five lasers (488 nm, 405 nm, 355 nm, 638 nm, and 561 nm) were used. UltraComp eBeads Plus Compensation Beads (Invitrogen) were prepared with single-antibody controls and used as single-color compensation controls. A randomly chosen donor blood sample was used for the Fluorescence Minus One (FMO) controls and isotype control (Rabbit IgG Isotype Control, AbBy Fluor 647 Conjugated, Bioss, Woburn, MA). HepG2, Jurkat, and genetically modified U937 cell lines were fixed, permeabilized, and stained with C4 antibody as negative and positive controls (*SI Appendix*, *Reagent Validation* and Figs. S1 and S2). *SI Appendix*, Fig. S4 shows the gating strategy used to identify the major immune cell populations: Neutrophils, natural killer (NK) cells, T cells, B cells, and monocyte subsets, specifically CD14^+^CD16^−^ [classical monocytes, (CM)], CD14^−^CD16^+^ [Nonclassical monocytes, (NCM)], and CD14^+^CD16^+^ (intermediate monocytes). Gates were determined using FMO controls. The Median Fluorescence Intensity (MFI) was obtained for the signal from the fluorescent tag conjugated to the C4 antibody for each donor.

### Unbiased Examination of C4 Gene Expression in Immune Cell Types Using Meta-Signature.

More than 50,000 immune cells have been previously analyzed in a meta-analysis of publicly available data (36). Briefly, effect sizes (Hedge’s g) were compared between samples from an immune cell subset of interest and all other immune cell subsets. The resulting gene expression was reported as a Standardized Mean Difference and the relative gene expression of a gene of interest in different immune cells (36). The Meta-Signature Tool (https://metasignature.stanford.edu/) was used to determine the immune cell types expressing C4A and C4B.

### The Clinical Comparison Cohort: Samples from Controls and Individuals with SZ.

Samples were collected from two studies: a *previously published Pilot Study* (24) and an *Expanded Cohort*. We referred to the two cohorts as the *Clinical Comparison Cohort*. The inclusion and exclusion criteria and sample handling for the Pilot Cohort were identical to those described for the Expanded Cohort, unless otherwise specified. Participant height and weight were measured to calculate the body mass index (BMI). Age, biological sex, and antipsychotic medication dosage were reported for each participant. Antipsychotic medication dosage was converted to olanzapine dose equivalents using the Defined Daily Dose Method for analyses (37).

#### Inclusion and exclusion criteria for the pilot and expanded cohorts.

Inclusion was limited to participants ages 18 to 35, but unrestricted with regard to ethnicity. Inclusion criteria for participants with SZ included a clinical DSM-V diagnosis of SZ or schizoaffective disorder, confirmation of one of these diagnoses by the Structured Clinical Interview for DSM-V (SCID-V), and initial diagnosis or initiation of antipsychotic medication within the last 5 y. Exclusion criteria included 1) a positive urine toxicology screen, as determined using a point-of-care urine screen (12panelNow, Boynton Beach, FL) which included screens for amphetamines, opiates, methamphetamines, methadone, benzodiazepines, cocaine, buprenorphine, 3-4 methylenedioxymethamphetamine, tetrahydrocannabinol, barbiturates, and phencyclidine; or 2) self-reported history of any of the following: current substance abuse or use of cannabis or tobacco products, a history of bleeding disorders, excessive bleeding with previous surgery, blood thinner use, autoimmune conditions, epilepsy, known genetic disorders, immunocompromised state (such as known diagnosis of Human Immunodeficiency Virus or Acquired Immunodeficiency Syndrome, taking prescribed or over the counter anti-inflammatories, chemotherapies or immunosuppressant agents), pregnancy, history of central nervous system disease, an uncontrolled medical disorder such as cancer, or inability to provide informed consent. The control participants were matched to the same age range as the group of individuals with schizophrenia, did not meet criteria for any DSM-5 disorder by the SCID-V, and had a Distress Score of less than or equal to 6 on the Prodromal Questionnaire-Brief Version (PQ-B) (38). The study was approved by the Stanford University Institutional Review Board and registered with Clinicaltrials.gov: NCT05109065. Informed consent was obtained from all study participants. Study data were collected and managed using REDCap electronic data capture tools hosted at Stanford University (39, 40).

#### Stress and psychosis symptom measures of study participants.

All enrolled participants were asked to complete a Perceived Stress Score Survey (PSS) (41). SZ participants were scored on the Positive and Negative Syndrome Scale (PANSS) by a trained interviewer (42).

#### Venous blood collection for the expanded cohort.

Approximately 50 mL of blood was collected in K2 EDTA blood collection tubes via venipuncture between 0800, and 1200 h from overnight fasting participants and processed on the same day. The samples were identified by sample number, blinding the scientists to the case information until the later stages of analysis. The protocols used in the Pilot Study were identical. The freezer storage time (FST) was determined by counting the number of days between sample collection and experimental analysis.

#### Plasma isolation and analysis.

Two milliliters of blood were centrifuged within 30 min after collection to isolate the plasma. Plasma was snap frozen in liquid nitrogen and stored at −80°C until measurement. Frozen plasma was allowed to thaw on ice and diluted in sample buffer (ProteinSimple, San Jose, CA) 1:500 at 4°C for measurement. Diluted plasma was exposed to C4 α-chain antibody (22233, RRID:AB_2879042, Proteintech, Rosemont, IL; targeted to both A and B forms of C4 protein) with capillary-system-based western WES Protein Simple (Bio-Techne, San Jose, CA). Plasma was diluted 1:500 and C4 antibody was used at 1:50 dilution. The samples were run in duplicate. Care was taken to balance controls and cases in each WES run (each cartridge held approximately 12 samples, analogous to the more familiar ‘gel’). The measured fluorescence intensity of the C4 α-chain (~90 kDa) was used to compare the C4 protein abundance in the samples. Purified C4 protein was used as a positive control in each run (C4 protein, Comptech, Tyler, TX). C4 protein abundance was normalized using the Sum Normalization method to reduce between-cartridge variability (43, 44).

#### Peripheral blood mononuclear cell (PBMC) collection for the expanded cohort.

Peripheral blood mononuclear cells (PBMC) were isolated from room-temperature whole blood using SepMate PBMC Isolation Tubes (StemCell Technologies, Seattle, WA). PBMCs were stored as live cells in liquid nitrogen in fetal bovine serum (FBS) with 10% dimethyl sulfoxide (DMSO). In a Pilot Study, PBMC samples were isolated using the traditional Buffy Coat Method and stored as live cells in liquid nitrogen in FBS with 10% DMSO (24).

#### Neutrophil clinical cohort: a subset of the clinical comparison cohort.

A subset of participant samples was processed for neutrophil isolation (Neutrophil Cohort, *SI Appendix*, Table S5*A*). The entire cohort was not included because of limited sample availability. Neutrophils were isolated from fresh whole blood using the EasySep Direct Neutrophil Isolation Kit (StemCell Technologies, Seattle, WA) and stored as cell pellets at −80°C. Pelleted cells were rinsed 1× with phosphate-buffered saline (PBS) and pelleted by centrifugation. Neutrophils were not stored as live cells (as PBMCs) because they are known to spontaneously degranulate during the freeze-thaw process (45).

#### Determination of the C4 genotype using digital droplet polymerase chain reaction.

Genomic DNA was isolated using standard techniques. The number of copies of the A, B, short (S) and long (L) forms of the C4 gene was determined as previously described using digital droplet polymerase chain reaction (ddPCR) (24, 25).

### Quantification of C4 Protein Associated with Distinct Immune Cells.

C4 protein was quantified in PBMCs using flow cytometry. Frozen live PBMC were thawed rapidly in 10% FBS in RPMI 1,640 media (Gibco, Thermo Fisher Scientific, Pittsburgh, PA) and then collected by centrifugation (300× g for 5 min at RT). The cells were resuspended in PBS and counted. Next, the cells were stained with the LIVE/DEAD Fixable Violet Dead Cell Stain Kit (Invitrogen, Waltham, MA) according to the manufacturer’s instructions. After washing with FACS buffer (1% bovine serum albumin and 0.1% sodium azide in PBS), the cells were incubated on ice for 15 min in blocking buffer (5% heat-inactivated AB human sera and 5% normal goat serum in PBS). Cells were then stained for exterior stains CD3, CD14, CD16, CD19, CD56, CD66b, CD45, and HLA-DR (same as those described above) by incubation for 30 min at RT in the dark. After washing twice with FACS buffer, cells were fixed and permeabilized using the BD Cytofix/Cytoperm Fixation/Permeabilization Kit (BD Biosciences, San Jose, CA). Finally, cells were stained for C4 protein (bs-15186R-BF647, Bioss, Woburn, MA) after antibody optimization by incubation in antibody in Perm/wash buffer. A parallel sample was stained with an isotype control (bs-0295P-BF647, Bioss, Woburn, MA). Similarly, FMO and compensation controls were freshly prepared for each experiment, as described above.

#### Neutrophil C4 protein measurement.

Neutrophils were isolated as previously described. Pelleted cells were rinsed 1× with phosphate-buffered saline (PBS) and pelleted by centrifugation. Cell pellets were resuspended in cold radioimmunoprecipitation assay buffer (RIPA; Millipore) and allowed to sit for 30 min to ensure cell lysis. Then, the lysates were flash-frozen in liquid nitrogen and thawed on ice. After centrifugation (20,000× g for 10 min at 4 °C), the lysate protein concentration was measured using a protein quantification assay (Pierce 600 nm Protein Assay Reagent with the addition of the Ionic Detergent Compatibility Reagent, Thermo Fisher, Pittsburgh, PA). Each samples’ lysate was run in duplicate under optimized conditions to measure abundance of C4 protein (0.5 mg/mL lysate concentration, 1:100 C4 antibody dilution) and actin (0.25 mg/mL lysate concentration, 1:250 actin antibody dilution (β-actin, 8H10B10, Invitrogen). The measured fluorescent intensity of the C4 α-chain, ~90 kDa, normalized by the actin loading control (at ~48 kDa) was used to compare the C4 protein abundance in samples from controls and individuals with SZ. Samples were further normalized using the Sum Normalization method to reduce between-cartridge variability (43, 44).

#### Classical and nonclassical monocyte isolation, immunofluorescent imaging and analysis.

A randomly selected subset of participant samples was processed for monocyte isolation (Monocyte Cohort, *SI Appendix*, Table S5*B*). Monocytes were separated from other PBMCs using the Pan Monocyte Isolation Kit (Miltenyi, Charlestown, MA) according to the manufacturer’s instructions. Then, CM (CD14^+^CD16^−^) were separated from NCM (CD14^−^CD16^+^) using CD14 Microbeads (Miltenyi, Charlestown, MA). The collected cells were then plated on gelatin-fibronectin-coated chamber slides (ibidi, Fitchburg, WI) in RPMI with 10% FBS. Cells were allowed to incubate at 37°C and 5% carbon dioxide for 30 min before being fixed with 4% paraformaldehyde in phosphate-buffered saline for 10 min. Fixed slides were stored at 4°C until they were stained with Hoechst 34580 (Invitrogen) and antibody directed against the C4 protein (22233-1-AP, Proteintech) using standard methods. Stained samples were imaged using a 63× oil-objective on a Zeiss LSM900 confocal microscope (Courtesy of the Erin Gibson Laboratory, Stanford University). All the samples were imaged with the same field size, exposure, and laser intensity. C4 protein fluorescence was measured across whole cells using a defined region of interest (ROI) in Fiji (46) using the mean fluorescent intensity (mean). The intensity measurements were averaged for the cells from each participant sample.

### Statistical Analyses.

The primary outcomes were the 1) group comparisons of C4 protein in different immune cells populations in the biobank samples and 2) correlation between Neutrophil and CM-associated C4 protein and the number of C4A gene copies (GCN) in the SZ and control samples. Benjamini–Hochberg procedure was used to correct for multiple tests (47). All other statistical tests were considered exploratory and were not corrected for multiple comparisons. Since immune cell-associated C4 protein and C4 gene copy frequencies were not normally distributed (Shapiro–Wilk test); nonparametric rank-based tests were used for all statistical analyses [two-tailed rank-based Spearman’s correlation and Kendall’s correlation for association analyses, Mann–Whitney *U* Tests for group comparisons and ANCOVA (Type II sums of squares) for group comparisons with covariates] (48). Confidence intervals were determined using bootstrapping, thus bias-corrected and accelerated (BCa) confidence intervals are reported (49, 50). Effect size, *r*, was calculated from the standardized test statistic, *Z*, obtained from the Mann–Whitney *U* Test. Partial eta-squared (η^2^_p_) was computed using stratified bootstrapping for group comparisons with covariates. Partial eta-squared (η^2^_p_) is the proportion of variance explained by that effect relative to the variance remaining after other factors are removed. We interpreted the effect size, *r*, and the rank-based correlation coefficient, *rho*, using conventional benchmarks, with values of approximately 0.20, 0.50, and 0.80 indicating small, medium, and large effects, respectively (Fig. 3*D*) (51–54). Likewise, we interpreted partial eta squared η^2^_p_ values of about 0.01, 0.06, and 0.14 as small, medium, and large effects, respectively (Fig. 3*D*). Statistical analyses were conducted in R within the RStudio integrated development environment using the ppcor, psych, boot, car, effectsize packages (48, 49, 55–60).

## Results

### C4 Protein Is Primarily Localized in Neutrophils and Monocytes.

**Fig. 1.**
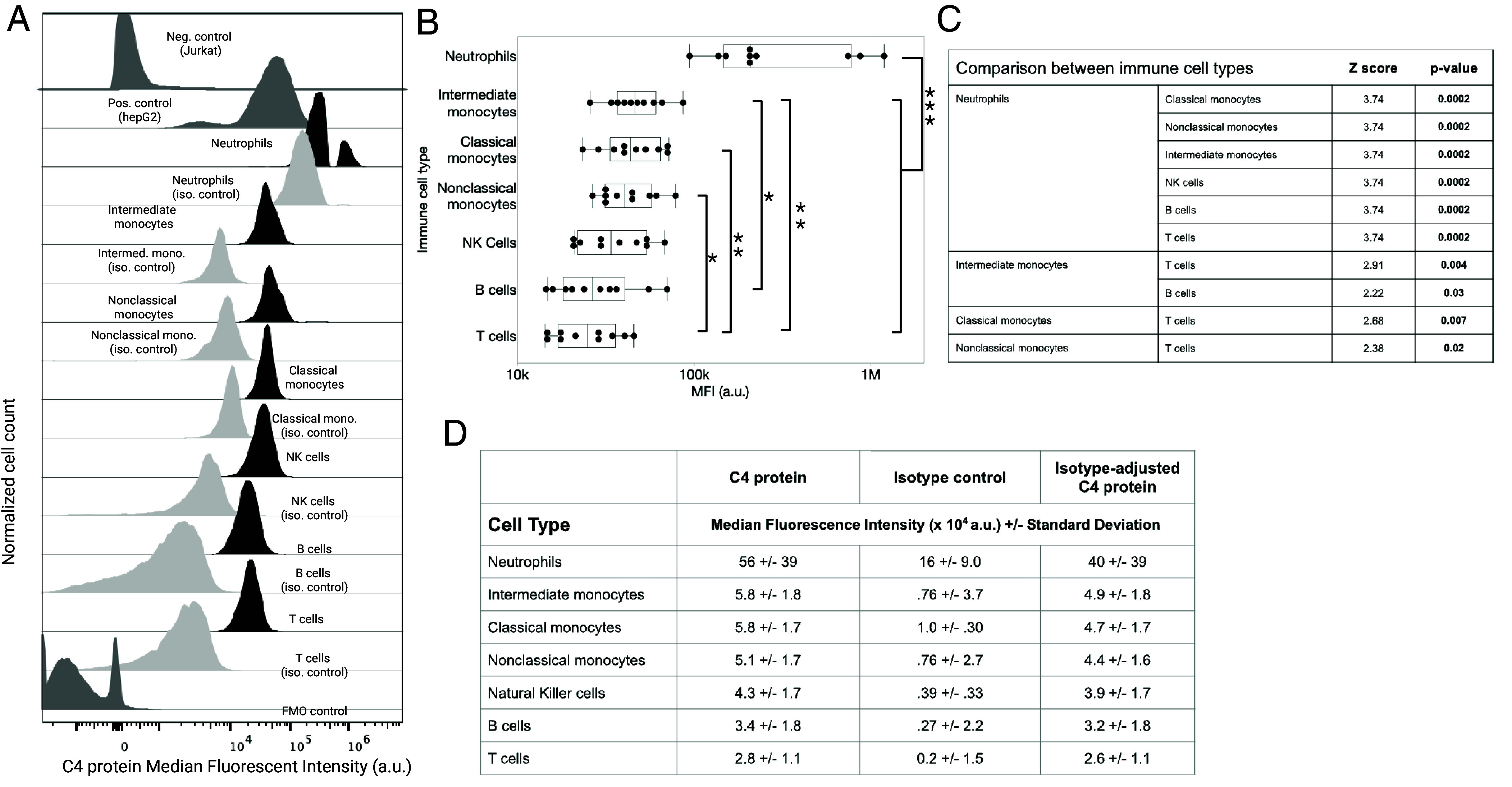
Neutrophils and monocytes express the C4A gene and contain C4 protein. (*A*) Flow cytometry of fresh whole-blood samples from anonymous donors. C4 protein was quantified (Median Fluorescent Intensity) in major immune cell types using flow cytometry. Jurkat cell line is the negative control and hepG2 is the positive control cell line (*Top* two panels in dark gray). Rabbit IgG isotype controls for each major immune cell type are shown in the panel below the C4 protein quantification in that immune cell type (light gray). The *Bottom* panel shows FMO control (dark gray). (*B*) MFI in major immune cell types in ten healthy donors from the Stanford Blood Center, as measured by flow cytometry. The MFIs were adjusted by subtracting the signal from the isotype control. Group differences between immune cell types were tested using the nonparametric Mann–Whitney *U* Test and significance was tested using the false discovery rate (FDR). * = *P* <0.05, ** = *P* <0.01, *** = *P* <0.001. (*C*) Comparisons that survived threshold for multiple comparisons are shown. A comprehensive list of comparisons is provided in *SI Appendix*, Table S3. (*D*) Average MFI of C4 protein signal in major immune cell types in 10 healthy donors from the Stanford Blood Center (*Left*), the isotype control values from the rabbit IgG isotype control for each immune cell type (*Middle*) and the isotype adjusted MFI (*Right*).

10 whole blood samples from anonymous donors were obtained from the Stanford Blood Center (demographic information in *SI Appendix*, Table S1). Using flow cytometry, we found C4 protein predominantly in neutrophils and all three types of monocytes (Fig. 1 *B* and *C*). The rabbit IgG isotype control signal was approximately 10 times lower than the measured C4 protein signal in all immune cell types, except for neutrophils (Fig. 1 *A* and *D*), suggesting that the antibody has increased nonspecific interactions with neutrophils compared to other immune cell types. The negative controls (FMO and Jurkat cell line, Fig. 1*A* and *SI Appendix*, Figs. S1 and S2) are lower in C4 protein MFI than all the immune cell types and isotype controls. The positive control cell line (hepG2, Fig. 1*A* and *SI Appendix*, Figs. S1 and S2) has a higher C4 protein MFI than all the isotype controls except for the neutrophil isotype control. The amount of C4 protein associated with neutrophils was higher than C4 protein associated with the other immune cells tested before (*SI Appendix*, Table S3) and after adjusting the MFI for cell type specific isotype controls (Fig. 1 *B* and *C*, *P* = 0.0002). Likewise, C4 protein associated with all three identified monocyte subtypes (CM, NCM, and intermediate) was higher than that in T cells (*P* = 0.007 for CM, *P* = 0.02 for NCM, and *P* = 0.004 for intermediate monocytes) (Fig. 1*C*). Intermediate monocyte cell MFI values were higher than those of B cells (*P* = 0.03).

Limited demographic information was available for the donors (*SI Appendix*, Table S1*A*). No significant correlations were observed between C4 protein MFI in the major immune cell types and age (*SI Appendix*, Table S1*B*).

### C4A and C4B Genes are Expressed in Different Cell Types.

C4A and C4B gene expression varied among different immune cell types (*SI Appendix*, Fig. S3 *A* and *B*). The C4A gene is expressed approximately twice as much in intermediate (CD14^+^CD16^+^) monocytes, neutrophils, specialized memory T cells, and dendritic cells as compared to other immune cells (*SI Appendix*, Fig. S3*A*). In contrast, the C4B gene was expressed at approximately three times the amount in T and B cells compared to other immune cells (*SI Appendix*, Fig. S3*B*). CM (CD14^+^) express less C4A and C4B genes compared to other immune cell types; however, they express twice the amount of the C4A gene compared to the C4B gene.

### Demographic and Clinical Characteristics of SZ and Control Participants.

A total of 38 controls and 25 individuals with SZ agreed to enroll in the study. Seven volunteers in the control group were ineligible after the SCID-V assessment due to a current psychiatric disorder. Three individuals with SZ were ineligible after SCID-V assessment due to meeting the criteria for a substance abuse disorder or not meeting the criteria for SZ or schizoaffective disorder. One control and two patients withdrew before completing the study. Thus, 30 healthy controls and 20 cases completed clinical evaluation and blood sampling (Table 1 and *SI Appendix*, Table S4). One control subject had to be excluded because of insufficient sample quantity. Additionally, nine controls and 10 individuals with SZ were included from our pilot study (24). All together, 38 control and 30 individuals with SZ were included in the analysis. The two groups did not differ significantly with respect to age, sex, or ethnicity. Individuals with SZ had a higher BMI than controls (BMI = 26.5 ± 5.2, in SZ and 23.9 ± 5.2 in controls, *P* = 0.02; Table 1), as is expected for this population (61). None of the control participants were taking any medications when enrolled in the study. All individuals with SZ were on medication: 97.2% were taking antipsychotic medication, 34.3% were taking more than one antipsychotic medication, 37% were taking antidepressants, 28.6% were taking medications for anxiety, and 8.6% were taking mood stabilizers. Additionally, 14.3% were taking benztropine and 2.9% were taking samidorphan.

**Table 1.** Demographics of the study participants in the Clinical Comparison Cohort

	Controls	Schizophrenia	*P*-value (Mann–Whitney *U* Test)
N of participants	38	30	
Mean Age ± SD	26.2 ± 5.1 y	24.6 ± 5.4 y	0.13
% Male (N)	58% (22)	67% (20)	0.62
Mean BMI	23.9 ± 5.2	26.5 ± 5.2	0.02
% European Ancestry (N)	58% (22)	60% (18)	–
% Black Ancestry (N)	3% (1)	7% (2)	–
% Asian Ancestry (N)	32% (12)	30% (9)	–
% Other Ancestry (N)	8% (3)	3% (1)	–
Ethnicity: % Hispanic (N)	13% (5)	20% (6)	–
PBMC FST (days)	1,070 ± 520	1,098 ± 656	0.54
Olanzapine dose equivalent (mg)	NA	8.6 +/− 14.1	–

Summary statistics for Age, Sex (percentage and number of male participants), BMI, PBMC Freezer Storage Time and Ethnicity are provided for control and individuals with SZ and Control groups. The distributions were compared using the Mann–Whitney *U* test. N = Number, SD = Standard Deviation, BMI = Body Mass Index, PBMC = Peripheral Blood Mononuclear Cells, FST = Freezer Storage Time. The Clinical Comparison Cohort consists of the Pilot and Expanded Cohorts together.

Subsets of the Clinical Comparison Cohort were used for neutrophil and monocyte experiments, based on sample availability. The demographics of the study participants comprising the Neutrophil and Monocyte Subsets are provided in *SI Appendix*, Table S5. They did not differ from the Clinical Comparison Cohort in terms of the metrics evaluated (age, sex, BMI, antipsychotic medication, FST, or ethnicity).

### Neutrophil C4 Protein Content Correlates with C4A Gene Copy Number in SZ Samples.

**Fig. 2.**
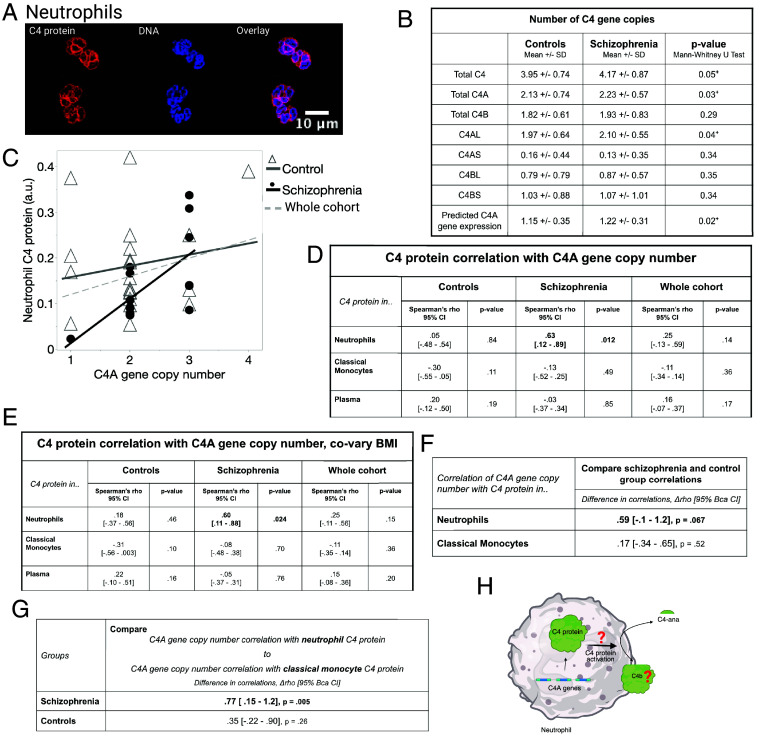
Neutrophil C4 protein is positively correlated with the number of C4A gene copies in SZ. (*A*) Isolated neutrophils from a healthy donor labeled for C4 protein [shown in red, labeled with anti-C4 antibody (Proteintech 22233)] and DNA (shown in blue, stained with DAPI). The overlay shows that the C4 protein is at the rim of the nuclear DNA. (*B*) The number of C4 gene copies for the various forms of the C4 gene (AS, AL, BS, and BL) was determined using ddPCR. Descriptive statistics for the number of each type of the C4 gene is provided for control and SZ groups. (*C*) Spearman correlation between measured neutrophil C4 protein and the number of C4A gene copies is provided for the control, SZ, and whole sample cohorts. The correlation between neutrophil C4 protein and the number of C4A gene copies was found to be statistically significant (*r* = 0.63, *P* = 0.012). (*D*) Table showing the Spearman’s correlations of the number of C4A gene copies and neutrophil-, classical monocyte (CM)- and plasma- C4 protein samples from SZ, control, and whole (combined SZ and control) cohorts. (*E*) Sensitivity analysis using BMI was a covariate in the partial correlation between the number of C4A gene copies and immune cell–associated C4 protein shows similar results. (*F*) The difference in correlation between neutrophil- and CM-associated C4 protein levels within each group (SZ and control samples analyzed separately), quantified using Fisher’s r-to-z transformation. (*G*) The between-group difference (SZ vs. control samples) in correlations between neutrophil- and CM-associated C4 protein and C4A gene copy number for each specified cell type. (*H*) Graphic illustrating C4A protein expression by C4A genes and subsequent activation. Whether the activation and consumption occurs inside neutrophils or the purpose of the activation is not known.

**Fig. 3.**
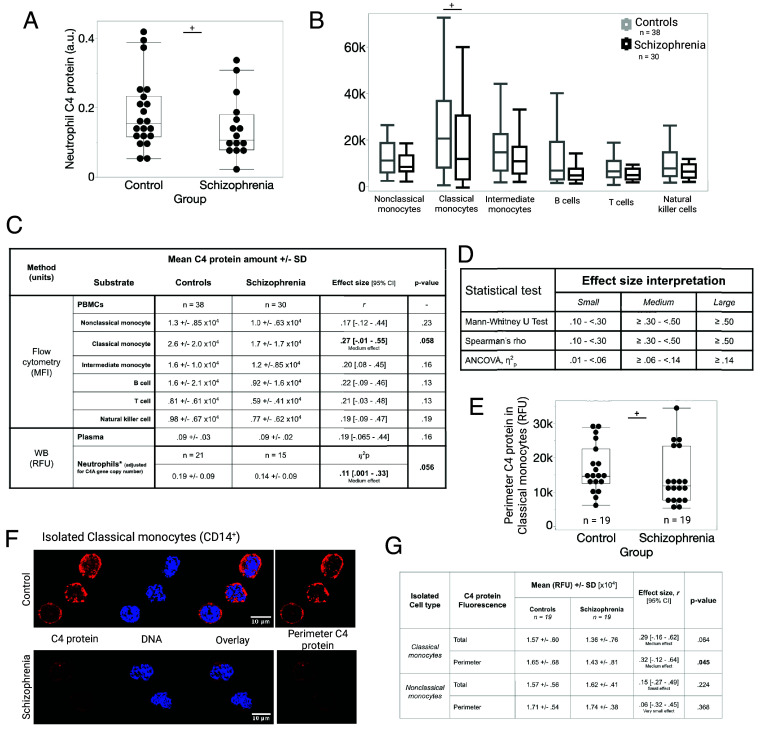
Exploratory analyses comparing the amount of C4 protein in different immune cell types between SZ and control samples. (*A*) C4 protein was measured by immunoblotting against C4 protein (22233 Proteintech, Rosemont IL) using WES (capillary-based western blotting) in isolated neutrophils from 15 SZ and 21 control blood samples. Samples were normalized using cellular actin (8H10D10, Invitrogen, Waltham, MA). (*B*) Flow cytometry was used to quantify C4 protein in major immune cell subtypes. (*C*) Table showing the descriptive statistics of measured immune cell-associated C4 protein in SZ samples compared to controls using different methods. Group comparisons of C4 protein for each major immune cell type (and plasma) were performed using the Mann–Whitney *U* Test. These same group comparisons were controlled for BMI using rank-based ANCOVA (*SI Appendix*, Table S8) (*D*) Effect size interpretation guidelines (Cohen’s conventional benchmarks). (*E–G*) CM and NCM were isolated from frozen live PBMC samples. Isolated cells were incubated, fixed, and stained for C4 protein (antibody directed against C4 protein, Proteintech, 22233-1-AP). C4 protein is localized throughout monocyte cells but is preferentially localized at the periphery. Representative images of CMs stained with Hoechst stain for DNA and fluorescently labeled antibody against C4 protein in control and SZ samples. (*F*) Quantification of C4 protein throughout each cell was performed by measuring the Mean Intensity (Mean) from immunofluorescent images of C4 protein using Fiji. A mask was created from the nuclear stain and used to subtract the central C4 protein fluorescence to determine the Peripheral C4 protein Mean Fluorescence. (*G*) Table showing the exploratory descriptive statistics of CM and NCM C4 protein in SZ and control samples. Exploratory one-way Mann–Whitney *U* Test was used to test differences between groups. Rank-based ANCOVA was used to control for BMI (*SI Appendix*, Table S9). RFU = Relative Fluorescent Unit.

We investigated whether C4 protein, (including A and B forms of C4 protein), within major immune cell types (neutrophils and monocytes) correlated with the number of C4A gene copies in SZ and/or control samples. To test this, we determined the number of C4A genes using ddPCR and C4 protein associated with neutrophils using western blotting, and in CM from live PBMCs using flow cytometry (Figs. 2 and 3). Correlations were tested using CM only, since the C4 protein associated with immune cells was similar in different monocyte subsets (Fig. 1 *B–D*), and CM are the dominant monocyte subtype in blood, constituting approximately 80% of total monocytes. In our cohort, we found a higher number of total C4, C4A, and C4AL gene copies in the SZ samples compared to the control samples (Fig. 2*B*). We found a strong positive correlation between the number of C4A gene copies and C4 protein only in neutrophils and only in the SZ samples that was statistical significance after correction for multiple tests (*df* = 13, *r_s_* = 0.63, 95% BCa CI: 0.12 to 0.89], *P* = 0.012, Fig. 2 *C* and *D*).

To assess the robustness of the relationship between C4A gene copy number and neutrophil C4 protein levels, we performed several sensitivity analyses. First, we confirmed our findings using Kendall’s tau, a more conservative correlation measure, which showed similarly strong results (*τ* = 0.55, 95% CI: (0.10 to 0.78), *P* = 0.011, *SI Appendix*, Table S6). Second, we evaluated the role of potential confounders. In control samples, neutrophil C4 protein correlated moderately with age (*r* = 0.46, *P* = 0.04, *SI Appendix*, Fig. S5 *A* and *B*) and BMI (*r* = 0.40, *P* = 0.07, *SI Appendix*, Fig. S5 *A* and *C*). However, when we adjusted for these variables in SZ samples, the correlation remained essentially unchanged: adjusting for BMI alone yielded a correlation coefficient similar to the original, *r* = 0.60 vs. original *r* = 0.63, (Fig. 2 *D* and *E*), likewise, adding BMI, age, sex, antipsychotic medication dosage, and FST to BMI as covariates yielded the same result, *r* = 0.60. This indicates that these factors do not meaningfully confound the relationship in SZ samples.

Third, excluding the SZ participant with one C4A gene copy and low neutrophil C4 protein still resulted in a strong correlation (*r* = 0.54, 95% BCa CI [0.02- *P* = 0.045). Finally, we compared neutrophil C4 protein levels between SZ and control participants who specifically had two copies of C4A. A Mann–Whitney *U* test on this subset (13 control sample mean = 0.17 ± 0.09; 8 SZ sample mean = 0.11 ± 0.04) revealed significantly lower neutrophil C4 protein in SZ samples, with a medium-to-large effect size (*df* = 21, *r* = 0.45, *P* = 0.039).

To determine whether the relationship between the number of C4A gene copies and neutrophil C4 protein is specific to SZ samples, we performed exploratory analyses that compared the correlations in immune cell types between control and SZ sample groups, and within groups between C4 protein in different immune cell types (Fig. 2 *F* and *G*). The difference in correlations between groups in neutrophil C4 protein was high (Δ*r* = 0.59, *P* = 0.067, Fig. 2*F*), though did not reach traditional significance. The difference in associations was robust even when controlling for BMI (Δ*r* = 0.51, *SI Appendix*, Table S7*A*). Furthermore, the correlation between neutrophil C4 protein and C4A gene copy number appears to be specific to neutrophil C4 protein; the comparison between neutrophil C4 protein and CM C4 protein (and their respective correlations with C4A gene copy number) was very strong [Δ*r* = 0.77, BCa CI (0.15 to 1.2), *P* = 0.005, Fig. 2*G*). Again, these correlational comparisons remained stable when covaried for BMI (Δ *r* = 0.76, *SI Appendix*, Table S7*B*).

### Exploratory Analysis: Decreased C4 Protein in Neutrophil and CM SZ Samples.

We conducted an exploratory analysis to compare whether the amount of C4 protein associated with specific immune cell types (neutrophils, NCM, CM, intermediate monocytes, B cells, T cells, and natural killer cells) and plasma differed between SZ samples and controls. C4 protein levels in plasma and neutrophils were compared using western blotting. C4 protein levels in other immune cell subtypes were compared using flow cytometry from previously frozen live PBMCs. C4A gene copy number was included as a covariate in the comparison of neutrophil C4 protein in control and SZ samples due to the strong association detected previously (Fig. 2 *B* and *C*).

We observed decreased C4 protein across *all* immune cells in SZ samples compared to controls (Fig. 3 *A–C*). The largest difference was in neutrophil C4 protein, [rank ANCOVA: F(1,33) = 3.92, η^2^_p_ = 0.11, medium effect size, 95% BCa CI (0.00, 0.33), *P* = 0.056, Fig. 3 *A* and *C*], with SZ samples showing lower neutrophil C4 protein after adjusting for C4A gene copy number. Unlike the correlational analyses, adding adjustment for BMI amplified the group difference between SZ and control samples, [rank ANCOVA: F(1,33) = 8.84, η^2^_p_ = 0.217, large effect size, 95% BCa CI (0.03, 0.46), *P =* 0.0055, *SI Appendix*, Table S8]. Notably, the 95% BCa CI in both calculations (with and without controlling for BMI) does not cross zero, which strengthens the evidence for the difference between groups.

CM C4 protein also showed a potential difference: [MFI = 2.59 ± 1.97 × 10^4^ in controls and 1.71 ± 1.70 × 10^4^ in SZ samples; Mann–Whitney *U* Test, *df* = 66, *r* = 0.27, small-medium effect size, 95% BCa CI (−0.01 to 0.55) *P* = 0.058, Fig. 3 *B* and *C*]. However, the difference decreased when BMI was added as a covariate, [rank ANCOVA: η^2^_p_ = 0.04, small effect size, 95% BCa CI (0.0003, 0.46), *P* = 0.10, *SI Appendix*, Table S8].

To further explore whether C4 protein associated with CM is altered in SZ samples, we isolated, plated, and stained CM (with NCM as a control) for C4 protein (Fig. 3*F*). The antibody we used for immunofluorescence was the same as the one we used for western blotting in evaluating neutrophil C4 protein abundance. However, we chose to perform immunofluorescence because it allowed us to determine the localization of C4 protein in CM and NCM. We observed that C4 protein was found at the perimeter of the cell and throughout the cytoplasm (Fig. 3*F*). Next, we compared the group means of total C4 protein (throughout the whole cell) and C4 protein at the perimeter, measured by the Mean Relative Fluorescent Intensity (RFU). We found a trend lower peripheral C4 protein RFU in SZ CM [1.43 ± 0.81 × 10^4^ in SZ samples vs. 1.65 ± 0.68 × 10^4^ in controls, Mann–Whitney *U* Test, *df* = 36, *r* = 0.32, medium effect size, 95% BCa CI (−0.12 to 0.45) *P* = 0.045, Fig. 3 *G* and *H*]. In contrast to the results using flow cytometry (Fig. 3 *B* and *C*), adjusting for BMI maintained the effect size of the difference between SZ and control samples and the 95% BCa CI does not cross zero, increasing the strength of the difference (rank ANCOVA: η^2^_p_ = 0.09, medium effect size, 95% BCa CI (0.00005, 0.33), *P* = 0.045, *SI Appendix*, Table S8). The pattern was similar for total (whole cell) C4 protein RFU in SZ CM compared to controls (1.36 ± 0.76 × 10^4^ in SZ samples vs. 1.57 ± 0.60 × 10^4^ in controls, *df* = 36, *r* = 0.29, medium effect size, 95% BCa CI (−0.16 to 0.62) *P* = 0.064, Fig. 3*G*; and rank ANCOVA: η^2^_p_ = 0.07, medium effect size, 95% BCa CI (0.0001, 0.29), *P* = 0.132, *SI Appendix*, Table S8) when adjusted for BMI. Conversely, we did not find a difference between SZ and control samples in NCM in either total or peripheral C4 protein RFU.

We examined the correlations between CM C4 protein and potential confounders (*SI Appendix*, Fig. S6). In CM C4 protein using flow cytometry, we find a small inverse correlation between CM C4 protein and FST (controls: *r* = −0.26, *P = 0.12*, SZ: *r* = −0.30, *P* = 0.10, and whole cohort: *r* = −0.32, *P* = 0.01, *SI Appendix*, Fig. S6 *A* and *B*). Conversely, we find a moderate positive correlation between CM C4 protein and FST in the SZ and whole cohort (SZ: *r* = 0.46, *P* = 0.05, and whole cohort: *r* = 0.32, *P* = 0.05, *SI Appendix*, Fig. S6 *C* and *D*).

### Exploratory Analysis: Neutrophil C4 Protein Associates Positively with Perceived Stress and SZ-Symptoms.

Additional exploratory analyses examined the association between quantifications of neutrophil- and CM- C4 protein with standardized measures of SZ-symptoms (PANSS) and perceived stress (PSS) (Table 2). The PSS measure was available for a subset of the sample while the PANSS measurements were available for all enrolled SZ participants.

**Table 2.** Exploratory correlational analyses between clinical measures and neutrophil- and classical monocyte- C4 protein

a				
Perceived Stress Score (PSS)				
	Sample size (n) Mean PSS ± SD	Mann–Whitney *U* Test Effect size, *r* [95% BCa CI]*P-value*	CM-C4 protein Spearman’s rho, [95% BCa CI] *P-value*	Neutrophil-C4 protein Partial Spearman’s rho, [95% BCa CI] *P-value*
Schizophrenia	n = 24 28.3 ± 5.9	0.69 (0.41 to 0.860) *P* < 0.001	n = 20 −0.17 (−0.59 to 0.29) *P* = 0.461	n = 13 0.53 (−0.11 to 0.87) *P* = 0.079
Controls	n = 30 20.4 ± 5.0		n = 28 −0.03 (−0.41 to 0.39) *P* = 0.879	n = 20 −0.34 (−0.73 to 0.21) *P* = 0.152
b				
**Perceived Stress Score** (Schizophrenia group only)	CM - C4 protein Spearman’s rho, (95% BCa CI) *P-value* n = 30	Neutrophil - C4 protein Partial Spearman’s rho, (95% BCa CI) P-value n = 15		
Total score	0.002 (−0.40 to 0.39) *P* = 0.992	0.33 (−0.34 to 0.77) *P* = 0.246		
Positive subscore	−0.14 (−0.54 to 0.25) *P* = 0.480	0.35 (−0.31 to 0.88) *P* = 0.226		
Negative subscore	0.16 (−0.22 to 0.52) *P* = 0.418	0.29 (−0.42 to 0.81) *P* = 0.313		
General psychopathology subscore	−0.04 (−0.46 to 0.35) *P* = 0.839	0.47 (−0.11 to 0.80) *P* = 0.090		

(*A* and *B*) Spearman correlational analyses were performed between cell-associated C4 protein (neutrophils and classical monocytes) and clinical symptom measures [Perceived Stress Score (PSS) in all study participants and Positive and Negative Syndrome Scale (PANSS) in participants with SZ]. Sample sizes, Spearman’s Correlation rho values, BCa confidence intervals, and uncorrected *P*-values are reported for each comparison. (*A*) Mean values for PSS in SZ and control groups are provided, along with uncorrected *P*-value from Mann–Whitney *U* Test. (*A* and *B*) Correlations involving neutrophil C4 protein were adjusted for the number of C4A gene copies using a Partial Spearman Correlation. Partial Spearman Correlations were also performed controlling for BMI only, and BMI and the number of C4A gene copies (*SI Appendix*, Table S10).

We found a statistically significant difference in the PSS between SZ and control groups, [mean = 27.2 ± 6.8, 20.4 ± 5.1, respectively, Mann–Whitney *U* Test, *r* = 0.69 (0.41 to 0.86), *P* < 0.001]. When examining the association between immune cell C4 protein and clinical measures, we observed a positive correlation between neutrophil C4 protein measurements and PSS in the SZ group [Spearman’s rho = 0.53, 95% BCa CI (−0.11, 0.87), *P* = 0.079, Table 2*A*], indicating a large effect size despite not reaching conventional statistical significance. Similarly, neutrophil C4 protein measurements showed a positive correlation with the PANSS general psychopathology subscale in the SZ group [*rho* = 0.47, 95% BCa CI (−0.11, 0.80), *P* = 0.09, Table 2*B*], also reflecting a large but not statistically significant effect. Both associations were unaffected by covarying for BMI (*rho* = 0.58 for PSS; 0.48 for PANSS general psychopathology subscore). Meaningful associations were not detected in the other groups.

## Discussion

The absence of treatments for schizophrenia underscores the need to better understand its underlying pathophysiology. Peripheral immune mechanisms represent a promising area for study. Treatment targets in the periphery are attractive because pharmaceutical agents do not need to cross the blood–brain barrier and still have therapeutic effects on the brain (19, 20). Innate immune mechanisms are a promising lead since they follow the disease course; particularly around C4 protein activation (C4-ana) (6, 11, 15, 30). In support of our overarching hypothesis, the presence of C4 protein in neutrophils and monocytes provides a nonplasma source of potential C4 protein activation in SZ. We demonstrated that C4 protein is expressed by and present in neutrophils and monocytes (Fig. 1 and *SI Appendix*, Fig. S3). Furthermore, when we compared the correlation between neutrophil C4 protein and the number of C4A gene copies, we found a large positive correlation *only* in the SZ group (Fig. 2). We did not find a correlation between C4 protein and the number of C4A gene copies for either group in CM or plasma. This association remained strong even after controlling for potential confounders (age, sex, BMI, FST, and antipsychotic medication) and the possible outlier. Likewise, we did not find a correlation between C4 protein and the number of C4B gene copies in neutrophils, CM, or plasma in either group.

Although these results are preliminary, they suggest potential clinical relevance: even after controlling for C4A gene copy number, neutrophil C4 protein correlated with clinical measures in individuals with schizophrenia. Stress exposure increases neutrophil and CM counts in both animal models and healthy human volunteers (62, 63). In patients with major depressive disorder, neutrophil and CM counts correlate positively with PSS (64). Since individuals with SZ show heightened stress sensitivity (65), we measured PSS in our cohort and explored its relationship with neutrophil- and CM- C4 protein in exploratory analyses. We captured the known stress sensitivity in SZ (Table 2*A*) (65). We also observed a large positive correlation between neutrophil C4 protein and PSS only in the SZ group (Table 2*A*). If confirmed in future studies, this finding would suggest that neutrophil C4 protein is not linked to a general stress experience—given the absence of an association in the control group, but rather to disease status. This interpretation is further supported by a moderate-to-large positive correlation between PANSS scores- particularly the general psychopathology subscore- and C4 protein exclusively in neutrophils (Table 2*B*). Although these findings are exploratory and have wide confidence intervals, this consistency is striking. We observed a similar positive correlation in the same cell type (neutrophil) using two distinct methods: self-reported questionnaire (PSS) and a clinician-administered interview (PANSS).

In additional exploratory analyses, we found neutrophil C4 protein to be lower in SZ samples than in controls (Fig. 3 *A* and *C*). This difference was even more pronounced after controlling for BMI (*SI Appendix*, Table S8). Because C4 protein appears to be expressed but not accumulated in SZ neutrophils, it may be consumed in a biological pathway or excreted (Fig. 2*H*). While we did not measure C4A and C4B gene expression directly in our study, the publicly available large dataset mRNA data indicate that neutrophils predominantly express C4A transcripts, so it is likely that the neutrophil C4 protein measured here is the A form (*SI Appendix*, Fig. S3). Taken together, these data suggest SZ neutrophils may actively express the C4A gene and protein. This interpretation is consistent with meta-analyses of cytokines in CHR, FEP, and chronic SZ which are elevated in SZ samples and are inducers of C4A gene expression, like interferon-γ and IL-6 (3, 66). We previously found a positive correlation between C4-ana (C4 protein activation product) and the number of C4A gene copies (24). Thus, we favor the interpretation that C4 protein is consumed in a biological pathway (Fig. 2*H*). If C4 protein was excreted, we would expect to find higher amounts of C4 protein in plasma in SZ samples because neutrophils make up about half (~40 to 60%) of all white blood cells in the peripheral circulation. We did not find higher amounts of C4 protein in plasma from SZ samples compared to controls, which is consistent with previous studies (Fig. 2). However, our assay measured total C4 protein (both C4A and C4B), so there may be a change in C4A protein concentration that is masked by the inclusion of C4B protein in our measurements. Further work is needed to test these hypotheses.

We also observed a modest decrease in C4 protein in SZ CM, particularly at the perimeter (Fig. 3*F*). While not statistically significant and uncorrected due to them being exploratory analyses, this trend was consistently observed using two different methods of measuring CM C4 protein and two different antibodies that target the C4 protein (Fig. 3 *A* and *D–F*). In contrast to the pattern observed for neutrophil C4 protein—with and without adjustment for BMI—the difference in CM C4 protein measured by flow cytometry decreased after controlling for BMI (Fig. 3*C* and *SI Appendix*, Table S8). Although the effect size was smaller, the confidence interval no longer crossed zero, suggesting a likely but modest difference in CM C4 protein between groups. The decrease in C4 protein was more pronounced at the cell perimeter (Fig. 3 *D–F*). However, the interpretation of decreased C4 protein in SZ CM is less clear. Similar to neutrophils, CM are also known to be in an activated state in SZ samples (67). However, we did not observe a correlation between the amount of C4 protein in CM and the number of C4A gene copies, suggesting that C4 protein is not actively expressed in CM. We also did not find a correlation with CM C4 protein and clinical measures. Thus, we hypothesized that C4 protein could be consumed as part of a low-grade CM activation in SZ samples. Additional studies are needed to test these hypotheses.

The detection of C4 protein inside neutrophils and monocytes is a novel basic finding. Our data suggest the existence of a C4 complosome, an intracellular C4 protein (36) (Fig. 2*H*). Neutrophils and monocytes express the C4A gene (*SI Appendix*, Fig. S3). However, the function of C4 protein in these immune cell types is unknown. We hypothesize that C4 protein is part of the biological pathways involved in neutrophil and monocyte activation because we see evidence of either consumption (in monocytes) and/or active transcription (in neutrophils) in SZ samples, which have been shown to be in an activated state (6, 68, 69) (Fig. 2*H*). Further research on the fundamental mechanisms underlying the function of C4 protein in neutrophils and monocytes, both in normal physiology and in the SZ disease context is warranted.

This study has several limitations. First, we used fresh whole blood, which required us to use blood donated on the day we performed the experiment (Fig. 1). Blood donors may have chronic conditions and are allowed to be on medication. The number of volunteers was small. In contrast, the C4 protein measured in the blood samples from biobank volunteers was largely in agreement with the gene expression data obtained from a much larger population (>800 samples, Fig. 1 and *SI Appendix*, Fig. S3). In our Clinical Comparison Cohort (Table 1), the interpretation of our study was limited by potential confounders such as medication use, FST, and high BMI in the SZ group. We did not detect a change in the correlation coefficient when the analysis was controlled for age, sex, BMI, antipsychotic medication, or FST. While we did not detect an association between either Neutrophil or CM C4 protein and medication use, this study should be repeated in samples from medication-naïve FEP that are known to progress to SZ to determine whether this association remains without the potential confounding of medication exposure. The associations that we detected between Neutrophil C4 protein and age and BMI were limited to the control group [associations between immune system changes in obesity and aging are well known (47, 48)], supporting the claim that the changes we found in Neutrophil C4 protein in SZ samples are more reflective of the disease state of SZ (*SI Appendix*, Fig. S5). In this study, β–actin was used as a loading control to quantify C4 protein in isolated neutrophils (Figs. 2 and 3). β–actin was chosen because it is commonly used in western blotting of human neutrophils (70–73). However, actin abundance may be altered in SZ samples (74). Future studies that quantify neutrophil C4 protein in SZ samples will need to perform single-cell measurements and/or look for protein in neutrophils that is unaffected by neutrophil activation or disease status. Furthermore, the strongest findings in our study arose from a small number of SZ neutrophil samples. Other studies that have examined the association between the number of gene copies and measured protein abundance find correlational coefficients in the range of 0.20 to 0.40 (52–54). Therefore, an association in the range of ~0.50 is considered a large effect size and likely meaningful.

In examining the potential associations between CM C4 protein and common confounders, we found a small negative correlation between CM C4 protein measured by flow cytometry and FST (*SI Appendix*, Fig. S6). Monocytes are known to be affected by freeze-thaw (75). The same small effect was observed in both the control and SZ groups. In the CM C4 protein measured by immunofluorescence, we observed a small negative correlation between CM C4 protein and FST in the SZ group (*SI Appendix*, Fig. S6). The fact that the directionality is opposite to that of CM C4 protein and FST measured by flow cytometry suggests that the association arises from the methods used to process the samples. CM were isolated prior to immunofluorescence, possibly selecting for a healthier subset of SZ CM. Selection of a healthier population of CM from SZ samples suggests that the loss of C4 protein associated with CM may be more pronounced than what we are able to measure in this study.

The finding of C4 protein in neutrophils (Fig. 1) and the positive association with the number of C4A gene copies (Fig. 2) support the hypothesis that the C4 protein in these cells could be the source of complement activation which is consistently observed in SZ samples (Fig. 2*H*). These findings integrate several previously disconnected aspects of innate immunity and observations in SZ that follow disease-related features. Specifically, they link: the risk association of SZ GWAS loci (number of copies of the C4A gene), evidence of complement activation in the plasma around the C4 protein, and increased counts and activation of primary innate immune cells (neutrophils and monocytes) (6, 11, 24, 25, 30, 69). Additionally, while excluded from our study cohort, individuals with SZ are well known to smoke cigarettes (76). Cigarette smoke is well known to activate neutrophils and monocytes and chronic smoking contributes to cognitive impairment, in individuals with SZ and controls (77–79). In contrast, it is well known that the most effective current medication for the treatment of SZ, clozapine, inhibits neutrophils (80, 81). Neutrophil elastase, an indicator of neutrophil activity, has been reported to be elevated in SZ (82). We have been able to suppress neutrophil-drive neuroinflammation with an elastase inhibitor, Silvelesat (83). Taken together, the convergence of these disparate factors strongly suggests that we may be honing a key pathophysiological mechanism of SZ pathophysiology that highlights the importance of neutrophils, which we hope will lead to accessible disease-altering therapeutics.

## Supplementary Material

Appendix 01 (PDF)

## Data Availability

Anonymized original data which includes flow cytometry data, absolute quantification of nucleic acids, images, Simple western spectra, symptom measures. data has been deposited in Stanford Digital Repository (DOI: 10.25740/vz984fc4200) (84).
