# Supplementary material for: Peripheral complement C4 protein in schizophrenia: Association with gene copy number and immune cell subtypes
Source: Proc Natl Acad Sci U S A. 2026 May 11;123(20):e2536376123. doi: 10.1073/pnas.2536376123 (PMC13187799; doi:10.1073/pnas.2536376123)
Supplement: Supplementary file 1 — Appendix 01 (PDF) [file pnas.2536376123.sapp.pdf]

Supplementary Materials

1. Reagent Validation

A. Identifying positive and negative control cell lines

To identify positive and negative controls for our experiments, we tested C4A/B mRNA expression and C4 protein abundance in common cell lines available in our laboratories.

Methods:

Measurement of C4A and C4B gene expression To determine mRNA expression, we grew cell lines (see Supplementary Figure 1a) according to standard ATCC protocols and collected cells using TRIzol reagent (Invitrogen, Waltham, MA, USA). Then, we isolated RNA using Direct-zol (Zymogen, Irvine, CA, USA). Reverse transcription was performed using the LunaScript Multiplex One-Step RT-PCR kit (New England Biolabs, Cambridge, MA) and C4A/B expression primers, as previously reported (1).

Measure C4 protein abundance. C4 protein abundance was measured using a capillary-electrophoresis-based system (ProteinSimple, San Jose, CA) and a commercially validated antibody directed against the alpha chain of the C4 protein (C4 Alpha Chain Polyclonal antibody; Proteintech, Rosemont, IL). Purified C4 protein was used as a positive control (CompTech, Tyler, TX, USA). Beta-actin was used as a loading control (Invitrogen, Waltham, MA, USA), and quantification of the alpha-chain peak was normalized to the actin peak.

Results:

The HepG2 and Jurkat cell lines were used as positive and negative controls, respectively. hepG2 (liver cancer cell line) produced significantly more C4A mRNA than the other cell lines tested. Among the cell

lines with low C4A/B gene expression, we chose the Jurkat cell line as our negative control cell line because it is derived from immune cells (T cells). C4 protein (alpha-chain) abundance was ~67 times higher in the hepG2 cell line than in the Jurkat cell line.

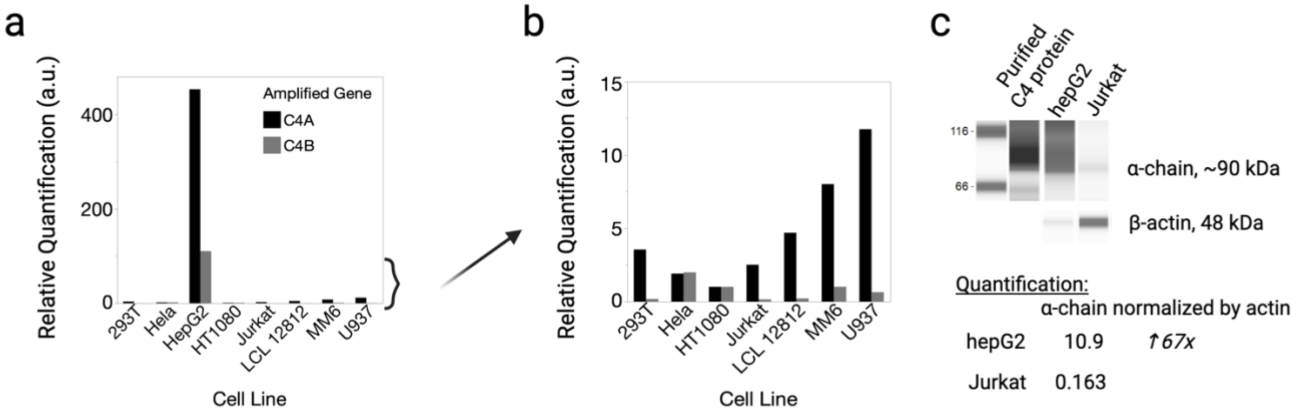

Supplementary Figure 1: (a) Quantitative reverse-transcription polymerase chain reaction of C4A and C4B gene expression in commonly available cell lines. The hepG2 (liver cancer cell line) produced significantly more C4A mRNA than the other cell lines tested. **(b)** Removing the hepG2 cell line, we observed the relative C4A and C4B mRNA expression in the other cell lines. Jurkat cell line was selected as the negative control cell line due to it being derived from immune cells (T cells). **(c)** ProteinSimple protein electrophoresis comparing hepG2 and Jurkat C4 protein (α-chain). Purified C4 protein was used as a positive control (CompTech, Tyler, TX, USA). Purified protein and cell line lysates were loaded based on protein quantification, however the a-chain around 90 kDa is overloaded by the close 120 kDa band (also a form of C4 protein) in the hepG2 and purified protein samples. Quantification of the a-chain (normalized by actin) indicates that the a-chain is 67 times more abundant in the hepG2 cell line compared to the Jurkat cell line.

B. <u>C4A gene knockdown</u>

To further validate that our antibody binds to the C4 protein, we created a U937 cell line with C4A/B gene knockdown.

Methods:

Genetic knockdown of C4A using the CRISPR-Cas9 System. The Gene Knockout Kit v2 was used for CRISPR-Cas9 knockdown of C4A gene (Synthego, Redwood City, CA). Single-guide RNAs (sgRNAs) selectively targeting C4A (and not C4B genes) were generated using the Synthego bioinformatics algorithm. Nucleofection was used to deliver sgRNA into the nuclei of the U937 cells (Lonza, Basel, Switzerland). After cell recovery, clonal dilution was performed to generate single-cell clonal lines according to Synthego's protocol for the clonal dilution of immortalized cell lines. Clonal lines with C4A gene knockdown were identified by protein screening. An abridged digital droplet polymerase chain reaction (determining the gene copy number of the A and B forms of the C4 gene) was performed to determine the number of copies of the C4A and C4B genes in the modified clonal lines (1).

The resulting C4A knockdown cell lines were used in flow cytometry experiments to confirm the specificity of C4 protein antibody binding. Cell lines were stained for viability (LIVE/DEAD Fixable Violet Dead Cell Stain Kit, Invitrogen, Waltham, MA) and C4 protein (Complement C4 polyclonal antibody, AbBy Fluor 647 Conjugated, Bioss, Woburn, MA). C4 protein MFI was determined only for live cells.

Results:

The wild-type U937 cell line was modified to generate a U937-C4 knockdown cell line. The single-guide RNA sequence used to target C4A for CRISPR knockdown is shown in Supplementary Figure 2a. The wild-type U937 cell line has five copies of the C4A gene (all five of which are the long form) and one copy of the C4B gene (short form). Digital-droplet PCR indicated that the U937 C4A knockdown lines had fewer copies of A and B forms of C4A (Supplementary Figure 2c). Protein screening showed a decrease in the abundance of the alpha chain (normalized to actin), with a decrease of approximately 87% after three rounds of CRISPR knockdown (in the $U937^{2A,0B}$ clonal line) compared to the *wild-type* $U937^{4A,1B}$ line

(Supplementary Figure 2b). Flow cytometry indicated that the antibody targeting the C4 protein showed a 40% decrease in C4 protein intensity (as indicated by the fluorescence signal at 647 nm) in the U937^{2A,0B} clonal line compared to the *wild-type* U937^{4A,1B} line (Supplementary Figure 2d). We suspect a lower magnitude of downregulation due to nonspecific binding of the rabbit polyclonal antibody. Since we observed an expected decrease in the signal when we decreased the number of C4A gene copies, we conclude that both antibodies appropriately target the C4 protein.

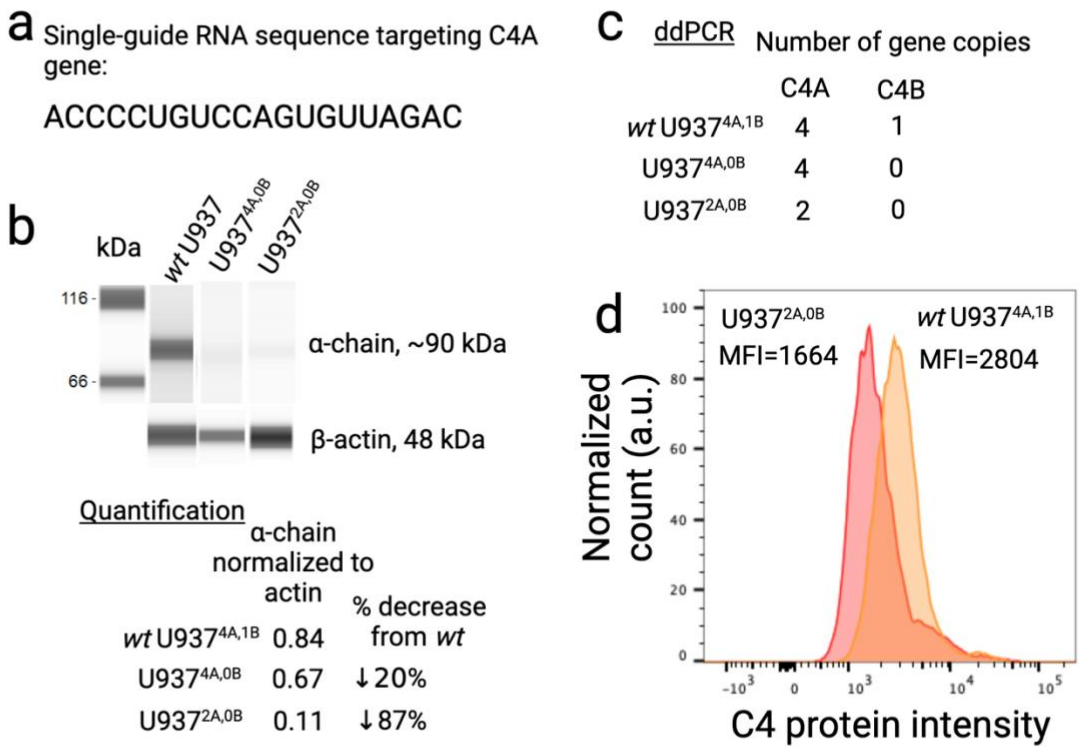

Supplementary Figure 2: (a) Single-guide RNA sequence used to target the C4A gene. **(b)** ProteinSimple protein electrophoresis comparing the *wild-type* U937 immortalized cell line to the first (U937^{4A,0B}) and third (U937^{2A,0B}) rounds of CRISPR knockdown of the C4A gene. In the third round of CRISPR knockdown, we observed a decrease in the alpha chain of the C4 protein. Quantification of the a-chain (normalized by the actin) indicates that the a-chain is decreased by approximately 87% in the third knockdown (U937^{2A,0B}). **(c)** Digital droplet polymerase chain reaction (ddPCR) was used to determine the number of gene copies of the C4A and C4B genes. We observed that C4B was disrupted after the first round of CRISPR. The subsequent CRISPR rounds disrupted the C4A genes, leaving only two intact C4A genes in the final clonal line (U937^{2A,0B}). **(d)** Fluorescence-activated cell

sorting indicated that the antibody targeting the C4 protein (Complement C4 Polyclonal Antibody, AbBy Fluor 647 conjugated, bs-15186R-BF647, Bioss, Woburn, MA) showed a 40% decrease in C4 protein intensity (as indicated by fluorescence signal at 647 nm wavelength) in the ($U937^{2A,0B}$) clonal line compared to the *wild-type* $U937^{4A,0B}$ line.

2. Supplementary Materials: Biobank Cohort

a

Biobank cohort	
N of participants	10
Mean Age +/- SD	51.6 +/- 17.9 years
Age range	31 - 79 years old
% Male (N)	50% (5)
% European Ancestry (N)	70% (7)
% Black (N)	0% (0)
% Asian (N)	20% (2)
% Other (N)	10% (1)
% Hispanic (N)	40% (4)

b

Correlation between C4 protein in immune cell type and age		
**Cell type**	**ρ**	**p-value**
Neutrophils	-0.05	0.89
Classical monocytes	-0.07	0.85
Nonclassical monocytes	-0.04	0.93
Intermediate monocytes	-0.08	0.83
NK cells	-0.03	0.93
T cells	0.10	0.78
B cells	0.10	0.77

Supplementary Table 1: Demographics of biobank volunteers. (a) Available demographic information on the volunteers who provided fresh whole blood samples on the day of the experiment. **(b)** Spearman's correlation between donor age and measured C4 protein levels associated with specific immune cell types.

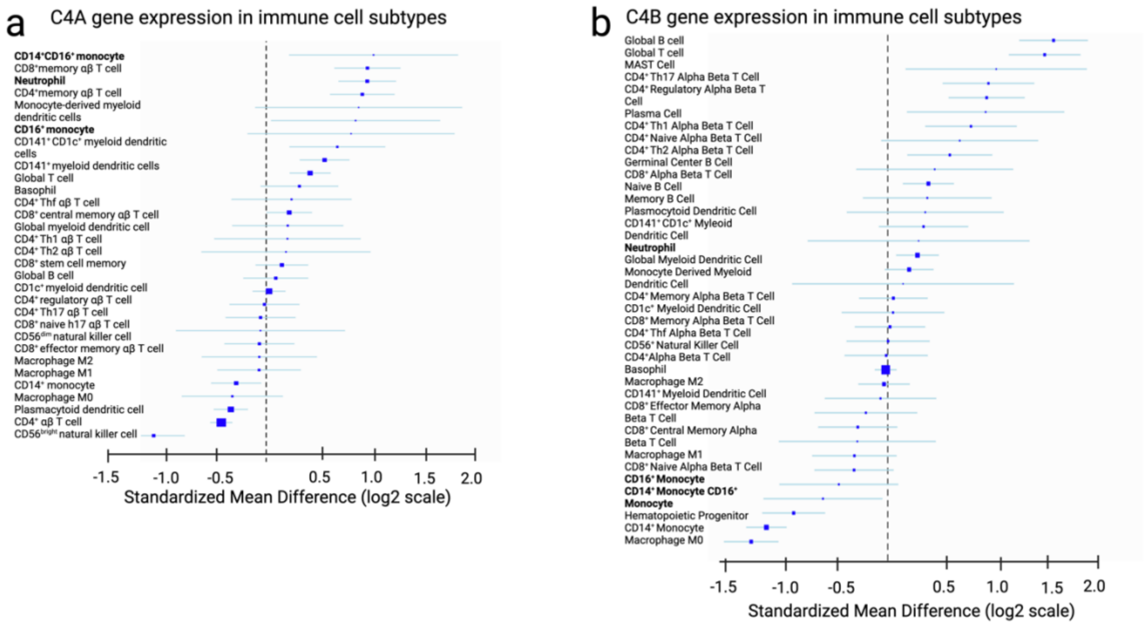

Supplementary Figure 3: Unbiased examination of C4 gene expression in immune cell types using (a-b) C4A gene expression and C4B gene expression in immune cell subtypes from Metasignature gui. More than 50,000 immune cells (and 853 available datasets) were previously analyzed in a meta-analysis of publicly available data, largely microarray data (2). Briefly, effect sizes (Hedge's g) were compared between samples from an immune cell subset of interest and all other immune cell subsets. The resulting gene expression was reported as a Standardized Mean Difference and the relative gene expression of a gene of interest in different immune cells (2). The Meta-Signature Tool (https://metasignature.stanford.edu/) was used to determine the immune cell types expressing C4A and C4B.

Target	Clone	Conjugated Fluorophore	Catalog number	RRID
CD3	UCHT1	BV605	300460	AB_2564380
CD14	M5E2	PE/Cy5	301864	AB_2860767
CD16	3G8	PE/Cy7	302016	AB_314216
CD19	HIB19	APC/Fire 810	302272	AB_2860771
CD45	HI30	BV711	304050	AB_2563466
CD56	NCAM16.2	BUV395	563554	AB_2687886
CD66b	G10F5	FITC	305104	AB_314496
HLA-DR	L243	PE	307606	AB_314684

Supplementary Table 2: Flow cytometry antibodies. The details for the flow cytometry antibodies used for the fresh whole blood and peripheral blood mononuclear cell experiments are listed here. All antibodies manufactured by BioLegend (San Diego, CA), except for CD56 (BD Biosciences, Franklin Lakes, NJ). RRID: Research Resource Identification.

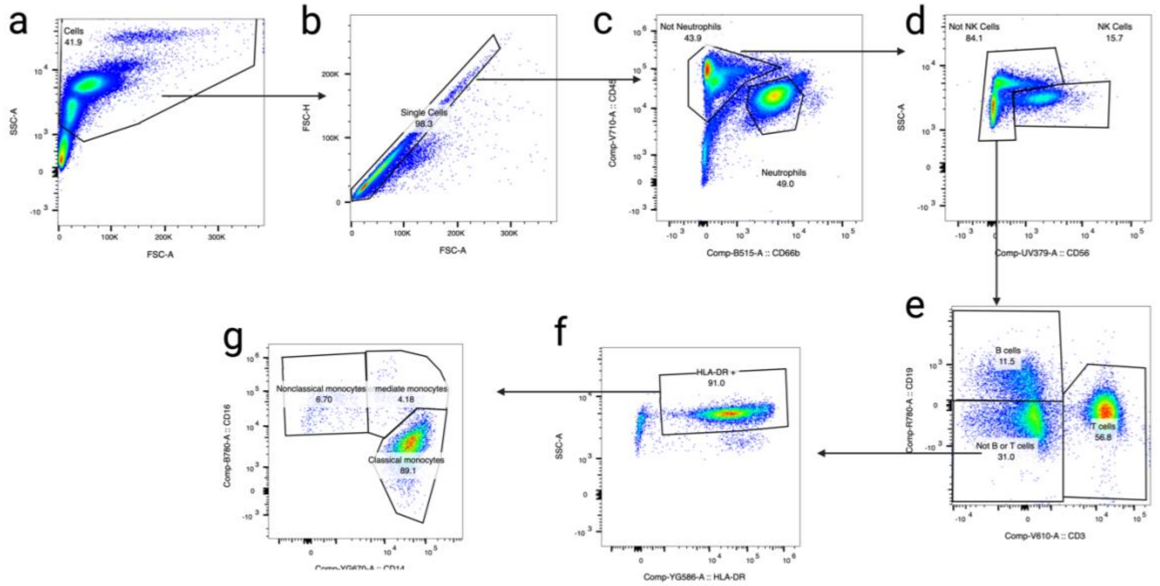

Supplementary Figure 4: Flow cytometry gating strategy. Flow cytometry gating of whole blood samples from donor 6 is provided as a representation of the gating strategy. Cells were gated: **(a)** first for lymphocytes based on size (area side-scatter and area forward scatter), then **(b)** for single cells by comparing the area and height of the forward scatter. **(c)** CD66b positivity and low CD45 expression in neutrophils. **(d)** Cells that were not neutrophils and were high in CD45 expression gated for Natural Killer (NK) cells using CD56 positivity. **(e)** Cells that were not NK cells were gated for T cells (CD3 positivity) and B cells (CD19 positivity). **(f)** True monocytes were gated using HLA-DR expression, **(g)** CD14 and CD16 positivity for monocyte subtypes. Specifically, $CD14^{+}CD16^{-}$ cells were defined as CM, $CD14^{-}CD16^{+}$cells as NCM, and $CD14^{+}CD16^{+}$ as Intermediate monocytes. Gates were determined using FMO controls. Compensation was performed using single fluorophore controls.

Comparison between immune cell types		Z score	p-value
Neutrophils	Classical monocytes	3.74	**0.0002**
Neutrophils	Nonclassical monocytes	3.74	**0.0002**
Neutrophils	Intermediate monocytes	3.74	**0.0002**
Neutrophils	NK cells	3.74	**0.0002**
Neutrophils	B cells	3.74	**0.0002**
Neutrophils	T cells	3.74	**0.0002**
Classical monocytes	B cells	2.6	**0.009**
Classical monocytes	T cells	3.28	**0.001**
Classical monocytes	NK cells	1.7	0.09
Classical monocytes	Nonclassical monocytes	0.86	0.38
Classical monocytes	Intermediate monocytes	0	1
Nonclassical monocytes	B cells	2.38	**0.01**
Nonclassical monocytes	T cells	2.98	**0.003**
Nonclassical monocytes	NK cells	1.02	0.3
Nonclassical monocytes	Intermediate monocytes	1.06	0.29
Intermediate monocytes	B cells	2.53	**0.01**
Intermediate monocytes	T cells	3.36	**0.0008**
Intermediate monocytes	NK cells	1.87	0.07
NK cells	T cells	2.07	**0.04**
NK cells	B cells	1.36	0.17
B cells	T cells	0.64	0.52

Supplementary Table 3: Group comparisons of C4 protein associated with different immune cells. Flow cytometry was used to quantify the amount of C4 protein associated with distinct immune cells. The resulting C4 protein Median Fluorescent Intensity (MFI), without subtracting the MFI value of the immune cell specific isotype controls, was compared for distinct immune cell types using the Mann-Whitney U test. A comparison is presented in this figure. Comparisons between monocyte

subtypes and B and T cells were omitted for clarity (clearly no difference). However, these were also included in the FDR analysis.

3. Supplementary Materials: Clinical Comparison Cohort

		Controls	Schizophrenia
Expanded Cohort	Telephone screened	69	55
	Consented for study	38	25
	Ineligible after SCID	7	3
	Withdrew from study	1	2
	Completed study	30	20
	Insufficient sample quantity	1	0
	**Total in Expanded Cohort**	**29**	**20**
	**Total from Pilot Cohort**	**9**	**10**
	**Clinical Comparison Cohort**	**38**	**30**

Supplementary Table 4: The “Clinical Comparison Cohort” contains samples from the previously collected “Pilot Cohort” (22) and the “Expanded Cohort.” Details of study recruitment, enrollment, and study completion of the Expanded Cohort are provided in the top portion of the table.

a

Neutrophil Subset	Controls	Schizophrenia	p-value (Mann-Whitney U Test)
N of participants	21	15	
Mean Age +/- SD	27.2 +/- 4.7 years	25.7 +/- 5.9 years	0.31
% Male (N)	48% (10)	52% (11)	0.39
Mean BMI	23.4 +/- 4.5	27.0 +/- 6.3	0.07
% European Ancestry (N)	48% (10)	73% (11)	-
% Black (N)	0% (0)	7% (1)	-
% Asian (N)	38% (8)	20% (3)	-
% Other (N)	14% (3)	0% (0)	-
% Hispanic (N)	14% (3)	13% (2)	-
Neutrophil FST (days)	789 +/- 300	632 +/- 464	0.46
OLZ dose equivalent (mg)	N/A	8.16 +/- 15.5	-

b

Monocyte Subset	Controls	Schizophrenia	p-value (Mann-Whitney U Test)
N of participants	19	19	
Mean Age +/- SD	25.6 +/- 5.4 years	25.5 +/- 5.7 years	0.86
% Male (N)	47% (9)	53% (10)	0.75
Mean BMI	23.4 +/- 6.7	26.4 +/- 6.2	0.08
% European Ancestry (N)	53% (10)	58% (11)	-
% Black (N)	11% (2)	5% (1)	-
% Asian (N)	37% (7)	32% (6)	-
% Other (N)	0% (0)	11% (1)	-
% Hispanic (N)	21% (4)	11% (2)	0.38
PBMC FST (days)	792 +/- 379	816 +/- 636	0.82
OLZ dose equivalent (mg)	N/A	9.6 +/- 14.1	-

Supplementary Table 5: Demographics of study participants for neutrophil (a) and monocyte (b) subsets. Summary statistics for Age, Sex (percentage of male participants and number of male participants), Body Mass Index (BMI), Plasma Freezer Storage Time, PBMC Freezer Storage Time and Ethnicity are provided for control and individuals with SZ and Control groups. The distributions were compared using the Mann-Whitney U test. Abbreviations: BMI = Body Mass Index, FST = Freezer Storage Time, N = Number, OLZ = olanzapine, PBMC = Peripheral Blood Mononuclear Cells, SD = Standard Deviation.

a

C4 protein correlation with C4A gene copy number						
C4 protein in..	**Controls**		**Schizophrenia**		**Whole cohort**	
	**Kendall τ 95% CI**	**p-value**	**Kendall τ 95% CI**	**p-value**	**Kendall τ 95% CI**	**p-value**
**Neutrophils**	.03 [-.41 - .46]	.81	**.55 [.10 - .81]**	**.01**	.21 [-.10 - .50]	.11
**Classical Monocytes**	-.06 [-.32 - .18]	.64	-.11 [-.43 - .20]	.46	-.08 [-.27 - .11]	.37
**Plasma**	.16 [-.10 - .41]	.18	-.03 [-.31 - .27]	.83	.12 [-.06 - .29]	.16

b

C4 protein correlation with C4A gene copy number, co-vary BMI						
C4 protein in..	**Controls**		**Schizophrenia**		**Whole cohort**	
	**Kendall τ 95% CI**	**p-value**	**Kendall τ 95% CI**	**p-value**	**Kendall τ 95% CI**	**p-value**
**Neutrophils**	.11 [-.33 - .47]	.48	**.52 [.10 - .78]**	**.01**	.21 [-.09 - .48]	.08
**Classical Monocytes**	-.08 [-.32 - .14]	.46	-.08 [-.41 - .28]	.56	-.09 [-.27 - .10]	.30
**Plasma**	.17 [-.09 - .40]	.12	-.04 [-.31 - .25]	.77	.12 [-.06 - .29]	.13

Supplementary Table 6: Kendall's correlation, a more conservative estimation of correlation. Kendall's correlation (τ, with confidence intervals) of neutrophil, monocyte and plasma with C4A gene copy number for samples from individuals with schizophrenia and controls with **(a)** no co-varies, and **(b)** co-varying with BMI.

a

Correlation of C4A gene copy number with C4 protein in..	**Compare schizophrenia and control group correlations** *Difference in correlations, Δrho [95% Bca CI]*
**Neutrophils**	.51 [-.27 - 1.3], p = .20
**Classical Monocytes**	.25 [-.81 - .31], p = .38

b

Groups	**Compare** *C4A gene copy number correlation with* ***neutrophil*** *C4 protein* *to* *C4A gene copy number correlation with* ***classical monocyte*** *C4 protein* *Difference in correlations, Δrho [95% Bca CI]*
**Schizophrenia**	**.76 [.04 - 1.5], p = .04**
**Controls**	.50 [-.13 - 1.1], p = .12

Supplementary Table 7: Comparison of Partial rank correlations, covarying for BMI. (a) Comparison between groups within immune cell type and **(b)** within each group between immune cell types.

a

Neutrophil (Western Blot)	Controls	Schizophrenia	Whole cohort
**Spearman's correlation of variable with measured C4 protein in neutrophils, ρ**			
Age	0.46, p = 0.04	0.09, p = 0.76	0.26, p = 0.12
Body Mass Index	0.40, p = 0.07	0.31, p = 0.26	0.26, p = 0.12
PBMC FST (days)	-0.20, p = 0.37	0.35, p = 0.20	0.11, p = 0.54
OLZ dose equivalent (mg)	NA	0.23, p = 0.42	-0.16, p = 0.36
Number of C4A gene copies	0.05, p = 0.84	0.63, **p = 0.01**	0.25, p = 0.14
Number of C4B gene copies	0.09, p = 0.70	-0.12, p = 0.67	-0.05, p = 0.77
**Compare means of reported participant sex with measured C4 protein in neutrophils, p-value**			
Reported Biological Sex	p = 0.97	p = 0.76	p = 0.72
Female	0.16 +/- 0.06	0.15 +/- 0.11	0.16 +/- 0.08
Male	0.20 +/- 0.13	0.14 +/- 0.08	0.17 +/- 0.11

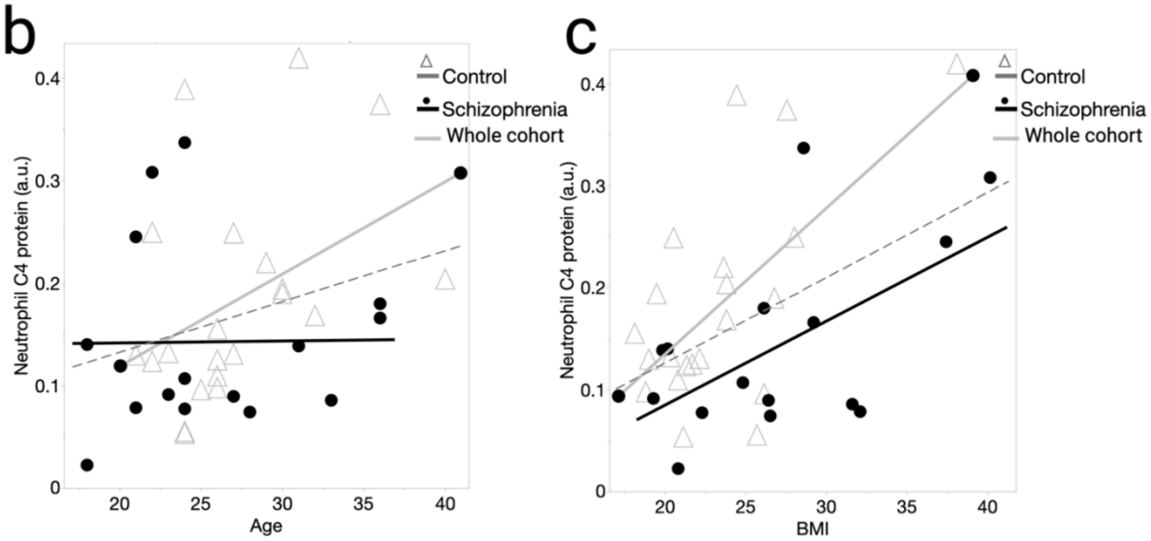

Supplementary Figure 5: Examining the role of potential confounders in measured neutrophil C4 protein abundance. (a) Table showing the analysis results Spearman correlation of neutrophil C4 protein abundance from flow cytometry and age, body mass index, PBMC freezer storage time (FST) in days and antipsychotic medication in olanzapine dose equivalents (OLZ dose equivalent) for the control group, SZ group and the combined (control and SZ) 'whole' cohort (except for OLZ dose equivalents which was only performed for the patient group). **(b-c)** Linear regression between neutrophil C4 protein abundance and **(b)** age and **(c)** body mass index (BMI).

a

Classical monocyte (Flow Cytometry)	Controls	Schizophrenia	Whole cohort
	Spearman's correlation of variable with measured C4 protein in CM, ρ		
Age	0.09, p = 0.58	0.22, p = 0.25	0.18, p = 0.14
Body Mass Index	-0.16, p = 0.33	-0.21, p = 0.27	-0.22, p = 0.07
PBMC FST (days)	-0.26, p = 0.12	-0.30, p = 0.10	-0.32, **p = 0.01**
OLZ dose equivalent (mg)	NA	0.13, p = 0.52	-0.13, p = 0.26
Number of C4A gene copies	-0.18, p = 0.30	-0.13, p = 0.49	-0.17, p = 0.18
Number of C4B gene copies	-0.03, p = 0.88	0.09, p = 0.62	0.01, p = 0.92
	Compare means of reported participant sex with measured C4 protein in CM, p-value		
Reported Biological Sex	p = 0.89	p = 0.71	p = 0.89
Female	2.54 +/- 2.15 x 10^4	1.69 +/- 1.37 x 10^4	2.22 +/- 1.90 x 10^4
Male	2.62 +/- 1.87 x 10^4	1.72 +/- 1.87 x 10^4	2.19 +/- 1.91 x 10^4

b

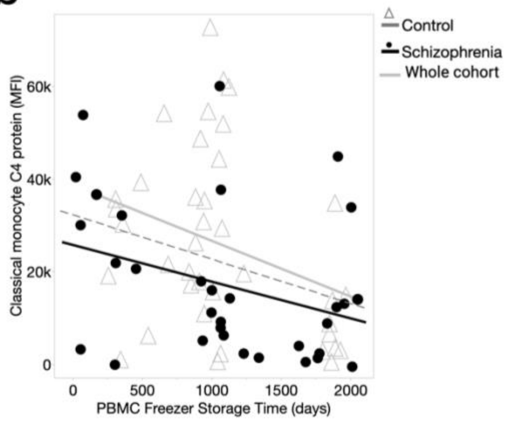

c

Classical monocyte (Immunofluorescence)	Controls	Schizophrenia	Whole cohort
	Spearman's correlation of variable with measured C4 protein in CM, ρ		
Age	0.18, p = 0.47	0.14, p = 0.56	0.14, p = 0.39
Body Mass Index	0.17, p = 0.49	0.23, p = 0.34	0.15, p = 0.38
PBMC FST (days)	-0.02, p = 0.93	0.46, **p = 0.05**	0.32, **p = 0.05**
OLZ dose equivalent (mg)	NA	0.36, p = 0.13	-0.13, p = 0.43
Number of C4A gene copies	-0.20, p = 0.42	-0.14, p = 0.57	-0.20, p = 0.22
Number of C4B gene copies	-0.02, p = 0.94	0.23, p = 0.35	0.07, p = 0.66
	Compare means of reported participant sex with measured C4 protein in CM, p-value		
Reported Biological Sex	p = 0.78	p = 0.97	p = 1.00
Female	1.65 +/- 0.66 x 10^4	1.34 +/- 0.65 x 10^4	1.51 +/- 0.65 x 10^4
Male	1.66 +/- 0.74 x 10^4	1.50 +/- 0.97 x 10^4	1.57 +/- 0.85 x 10^4

d

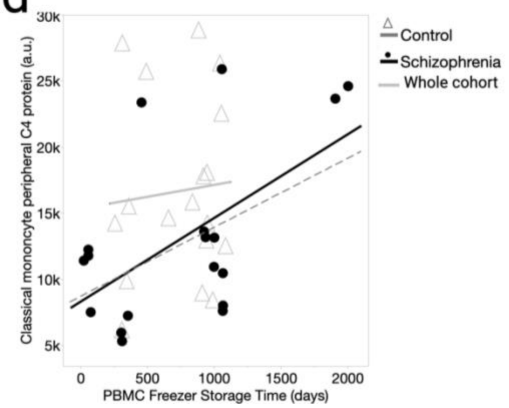

Supplementary Figure 6: Examining the role of potential confounders in measured classical monocyte C4 protein abundance. **(a)** Table showing the analysis results Spearman correlation of CM C4 protein abundance from flow cytometry and age, body mass index, PBMC freezer storage time (FST) in days and antipsychotic medication in olanzapine dose equivalents (OLZ dose equivalent) for the control group, SZ group and the combined (control and SZ) 'whole' cohort (except for OLZ dose equivalents which was only performed for the patient group). **(b)** Linear regression between flow cytometry-determined C4 protein abundance in CM and PBMC freezer storage time. **(c)** Table showing the analysis results Spearman correlation of CM C4 protein abundance from immunofluorescence and age, body mass index, PBMC freezer storage time (FST) in days and antipsychotic medication in olanzapine dose equivalents (OLZ dose equivalent) for the control group, SZ group and the combined (control and SZ) 'whole' cohort (except for OLZ dose equivalents which was only performed for the patient group). **(d)** Linear regression

between Immunofluorescence-determined C4 protein abundance in CM and PBMC freezer storage time for the control group, SZ group and the combined (control and SZ) 'whole' cohort.

Method (units)	Mean C4 protein amount +/- SD, covary for BMI				
	**Substrate**	**Controls**	**Schizophrenia**	**Effect size** ($\eta^2 p$) [95% CI]	**p-value**
Flow cytometry (MFI)	**PBMCs**	n = 38	n = 30	-	-
	**Nonclassical monocyte**	1.3 +/- .85 $\times 10^4$	1.0 +/- .63 $\times 10^4$	.012 [0 - .109]	.38
	**Classical monocyte**	2.6 +/- 2.0 $\times 10^4$	1.7 +/- 1.7 $\times 10^4$	.041 [.0003 - .19] Small effect	.10
	**Intermediate monocyte**	1.6 +/- 1.0 $\times 10^4$	1.2 +/-.85 $\times 10^4$	.017 [0 - .118]	.29
	**B cell**	1.6 +/- 2.1 $\times 10^4$	.92 +/- 1.6 $\times 10^4$	.023 [.0001 - .138]	.23
	**T cell**	.81 +/- .61 $\times 10^4$	.59 +/- .41 $\times 10^4$	.024 [.0001 - .148]	.21
	**Natural killer cell**	.98 +/- .67 $\times 10^4$	.77 +/- .62 $\times 10^4$	.014 [0 - .12]	.34
WB (RFU)	**Plasma**	.09 +/- .03	.09 +/- .02	.016 [0 - .112]	.26
	**Neutrophils*** (adjusted for C4A gene copy number and BMI)	n = 21	n = 15	.22 [.03 - .46] Large effect	.0055
		0.19 +/- 0.09	0.14 +/- 0.09		

Supplementary Table 8: Comparison of immune cell C4 protein in SZ and control samples while controlling for BMI. Table showing the mean C4 protein abundance in different immune cell samples and the effect size, (η^2p), of the rank-based ANCOVA comparing them.

Isolated Cell type	C4 protein Fluorescence	Mean (RFU) +/- SD [x10^4] Covary for BMI		Effect size (η^2p) [95% CI]	p-value
		Controls *n = 19*	Schizophrenia *n = 19*		
Classical monocytes	Total	1.57 +/- .60	1.36 +/- .76	.07 [.0001- .29] Medium effect	.132
	Perimeter	1.65 +/- .68	1.43 +/- .81	.09 [.00005 - .33] Medium effect	**.045**
Nonclassical monocytes	Total	1.57 +/- .56	1.62 +/- .41	.016 [0 - .65] Small effect	.334
	Perimeter	1.71 +/- .54	1.74 +/- .38	.003 [-.32 - .45] Very small effect	.192

Supplementary Table 9: Exploratory comparison of immune cell C4 protein in SZ and control samples while controlling for BMI. Table showing the mean C4 protein abundance in classical and nonclassical monocytes measured by immunofluorescence. Comparisons between SZ and control samples were made using the non-parametric rank-based ANCOVA controlling for BMI. Effect size, (η^2p), with BCa 95% confidence interval obtained by bootstrapping are reported.

a

	Covary BMI only		Covary BMI and C4A gene copy number	
**Perceived Stress Score**	**CM-C4 protein** MFI (a.u.)	**Neutrophil-C4 protein** WB (a.u.)	**CM-C4 protein** MFI (a.u.)	**Neutrophil-C4 protein** WB (a.u.)
Schizophrenia	n = 20<br>rho = -.19 [-.62 - .28]<br>*p = .426*	n = 13<br>rho = .27[-51 - .79]<br>*p = .389*	n = 20<br>rho = -.25 [-.63 - .32]<br>*p = .343*	n = 13<br>rho = .58 [-27 - .88]<br>*p = .064*
Controls	n = 28<br>rho = -.04 [-.46 - .42]<br>*p = .849*	n = 20<br>rho = -.20 [-.63 - .29]<br>*p = .407*	n = 28<br>rho = .04 [-.42 - .50]<br>*p = .837*	n = 20<br>rho = -.32 [-.75 - .29]<br>*p = .192*

b

Spearman's correlational analyses (rho)	Covary BMI only		Covary BMI and C4A gene copy number	
	**CM-C4 protein** MFI (a.u.)	**Neutrophil-C4 protein** WB (a.u.)	**CM-C4 protein** MFI (a.u.)	**Neutrophil-C4 protein** WB (a.u.)
**Positive and Negative Syndrome Scale** (Schizophrenia group only)	**n = 30**	**n = 15**	**n = 30**	**n = 15**
Total score	.02 [-.41 - .45]<br>*p = .908*	.33 [-.24 - .76]<br>*p = .253*	.02 [-.44 - .40]<br>*p = .942*	.33 [-.37 - .81]<br>*p = .264*
Positive subscore	-.11 [-.53 - .32]<br>*p = .601*	.28 [-.34 - .77]<br>*p = .331*	-.12 [-.54 - .31]<br>*p = .554*	.31 [-.50 - .84]<br>*p = .298*
Negative subscore	.17 [-.22 - .54]<br>*p = .394*	.33 [-.34 - .82]<br>*p = .252*	.18 [-.32 - .54]<br>*p = .380*	.33 [-.41 - .85]<br>*p = .270*
General psychopathology subscore	-.03 [-.48 - .40]<br>*p = .898*	.30 [-.39 - .82]<br>*p = .294*	-.06 [-.46 - .30]<br>*p = .777*	.44 [-.20 - .84]<br>*p = .132*

Supplementary Table 10: Exploratory correlation between neutrophil- and CM- C4 protein with clinical measures, (a) PSS and (b) PANSS. Partial Spearman correlations were calculated for clinical measures with biological (neutrophil and CM C4 protein) both covarying for BMI only and BMI and the number of C4A gene copies. Confidence intervals were generated with bootstrapping and given as BCa confidence intervals.

Supplementary Materials References

1. A. Sekar, *et al.*, Schizophrenia risk from complex variation of complement component 4. *Nature* **530**, 177–183 (2016).

2. F. Vallania, *et al.*, Multicohort Analysis Identifies Monocyte Gene Signatures to Accurately Monitor Subset-Specific Changes in Human Diseases. *Front. Immunol.* **12**, 659255 (2021).
